# The proppin Bcas3 and its interactor KinkyA localize to the early phagophore and regulate autophagy

**DOI:** 10.1080/15548627.2020.1725403

**Published:** 2020-03-01

**Authors:** Yoko Yamada, Pauline Schaap

**Affiliations:** a School of Life Sciences, University of Dundee, Dundee, UK; b Department of Biology, Faculty of Science, Toho University, Funabashi, Japan

**Keywords:** Atg8 processing, breast carcinoma amplified sequence 3, *Dictyostelium*, inositol phospholipid binding, Knka, phagophore assembly

## Abstract

To resolve the signaling mechanisms that mediate the starvation-induced processes of *Dictyostelium* sporulation and encystation, we performed insertional mutagenesis on cells harboring an mRFP-tagged spore gene. We isolated a mutant in *kinkyA* (*knkA*), a gene without known function, which formed fruiting bodies with a kinked stalk and lacking viable spores. Immunoprecipitation of lysates of KnkA-YFP-transformed *knkA^−^* cells yielded a mammalian BCAS3 homolog as a KnkA interactor. *bcas3^−^* phenocopied *knkA^−^* and Bcas3 colocalized with KnkA to puncta. Bcas3 shares sequence similarity with proppins (beta-propellors that bind phosphoinositides). Mutation of 2 Bcas3 residues that are essential for PtdIns3P binding in proppins prevented Bcas3 binding to PtdIns3P as well as punctate Bcas3 and KnkA localization. KnkA puncta also colocalized with small but not large vesicles that contain the autophagy protein Atg8 and were contiguous with the endoplasmic reticulum. *knkA^−^* and *bcas3^−^* cells showed a pronounced decrease of RFP-GFP-Atg8 in neutral early autophagosomes, indicating that KnkA and Bcas3 are required for macroautophagy/autophagy. Knockouts in *atg7, atg5* or *atg9* substantiated this finding by showing similar sporulation defects as *knkA^−^* and *bcas3^−^*. Defective *Dictyostelium* sporulation is evidently a useful diagnostic tool for the discovery of novel autophagy genes.

**Abbreviations:** Atg: Autophagy-related; BCAS3: BCAS3 microtubule associated cell migration factor; cAMP: 3ʹ,5ʹ-cyclic adenosine monophosphate; ER: endoplasmic reticulum; GFP: green fluorescent protein; PAS: phagophore assembly site; PRKA/PKA: protein kinase cAMP-dependent; Proppin: beta‐propellers that bind phosphoinositides; PtdIns3P: phosphatidylinositol 3-phosphate; REMI: restriction enzyme-mediated insertional mutagenesis; RFP: red fluorescent protein; RT-qPCR: reverse transcriptase - quantitative polymerase chain reaction; WIPI: WD repeat domain, phosphoinositide interacting; YFP: yellow fluorescent protein

## Introduction

The differently phosphorylated forms of the inositol phospholipids play a major role in recruiting proteins to different membrane compartments. They control processes such as exo-, endo- and phagocytosis and autophagy, as well as cell polarity, migration, and cell division. The activities of a range of phosphorylation-site specific phosphatidylinositol (PtdIns) kinases and phosphatases dynamically generate a total of 7 mono-, bis- and tris PtdIns phosphates that are differentially enriched in specific membranes at specific times. Overall, PtdIns(4,5)P_2_ and PtdIns(3,4,5)P_3_ are typically part of plasma membranes, PtdIns3P is enriched in endosomes and autophagosomes and PtdIns4P typifies Golgi membranes, but neither phosphatidylinositol phosphate is unique to these membranes (see [1] for a review).

The target proteins for recruitment to different membrane compartments contain PtdInsP binding domains, such as the PH domains that bind to either PtdIns(4,5)P_2_ or PtdIns(3,4,5)P_3_, the ENTH and ANTH domains that also bind PtdIns(4,5)P_2_, the FYVE domain that binds PtdIns3P and the PX domain that bind PtdIns3P and PtdIns(3,4)P_2_. Proppins (beta-propellers that bind phosphoinositides) are members of a larger family of proteins with 7 WD40 domains, that form a 7-bladed propeller. Such proteins have a general role in protein-protein interactions [2], but the proppins additionally bind 2 molecules of PtdIns3P and/or PtdIns(3,5)P_2_, using conserved amino-acids in blades 5 and 6 of the propeller [3].

The well-studied yeast Hsv2 (Ygr223c), Atg18 and Atg21 proppins all have roles in autophagy. Hsv2 functions in micronucleophagy [4]. Atg21 recruits Atg8 and Atg16 to the assembly site for the phagophore [5], the double-membrane structure that will enclose cellular components in nonselective autophagy. This recruitment enables the ligation of Atg8 to phosphatidylethanolamine, an essential step in phagophore formation and expansion. Atg18 is required for both selective cytoplasm to vacuole targeting (Cvt) autophagy [6] and for nonselective autophagy in yeast, where a complex of Atg18 and Atg2 binds to PtdIns3P and the integral membrane protein Atg9 at the ends of the phagophore. Atg2 forms a tether between the phagophore and the ER, possibly to facilitate lipid transport to the expanding phagophore [7,8]. Atg18 carries membrane scission activity, which is hypothesized to play a role in lipid transport between compartments [9]. Mammals have 4 proppin homologs WIPI1, WIPI2, WDR45B/WIPI3 and WDR45/WIPI4, of which WIPI1 and WIPI2 are similar to Atg18 and WDR45B and WDR45 are similar to Hsv2 [10].

We investigate mechanisms that control sporulation in *Dictyostelium* fruiting bodies using insertional mutagenesis of cells expressing an mRFP-tagged spore coat protein. We identified a mutant that makes fruiting bodies with kinked stalks and few viable spores. The genetic lesion occurred in a deeply conserved gene of unknown function, which was named *KinkyA* (*knkA*), and we identified a homolog to another deeply conserved protein, Bcas3, as a KnkA-interacting protein. Both *bcas3* and *knkA* knockouts showed the same morphological phenotype, which was similar but somewhat weaker than that created by knockout of essential autophagy genes such as *atg7, atg5*, or *atg9* [11]. Bcas3 proved to be a proppin that required its inositol phospholipid-binding domain to localize to the *Dictyostelium* phagophore assembly site (PAS) and also to recruit KnkA to the PAS. *KnkA* and *bcas3* knockouts showed a reduction of early autophagosomes compared to wild-type cells. We conclude that KnkA and Bcas3 are novel positive regulators of the autophagic machinery.

## Results

### A protein with an UPF0183 domain is essential for spore differentiation

**Figure 1.**
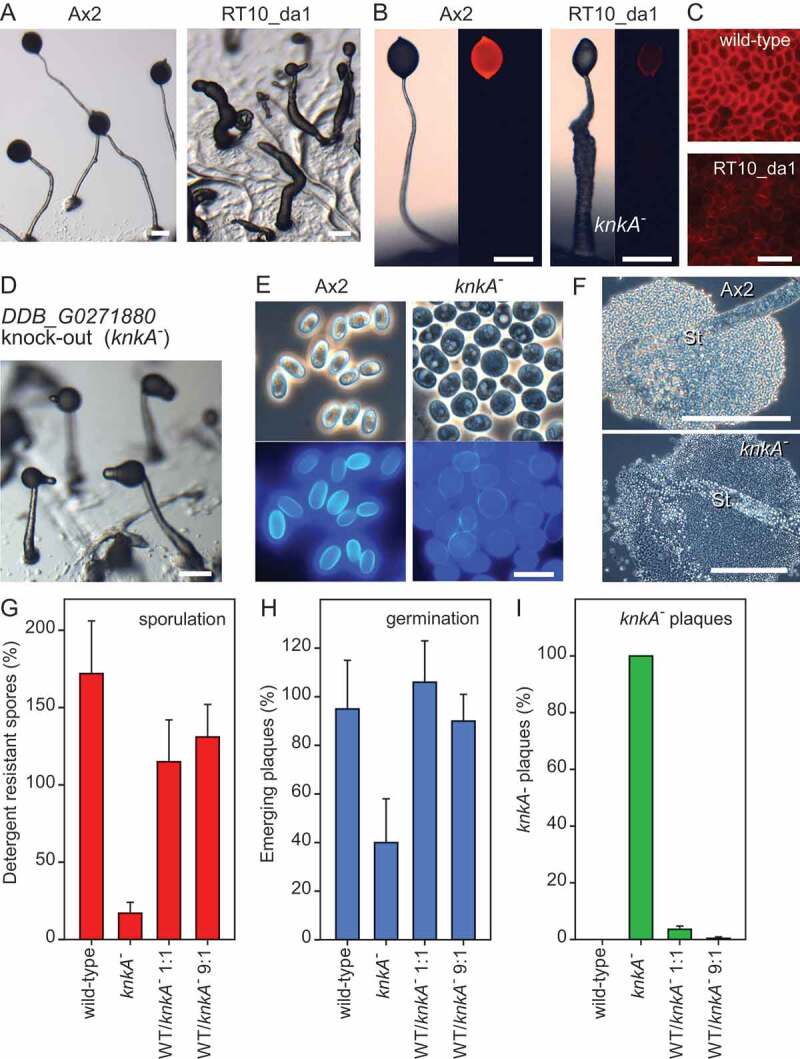
The phenotype of REMI clone RT10_da1 and a DDB_G0271880 (*knkA*) knockout. (A-C) REMI mutant phenotype. (A) Cells of the parent Ax2, transformed with CotC-mRFP, and REMI mutant RT10_da1 were developed into fruiting bodies and photographed. (**B/C)**. Fruiting bodies and spores were imaged under epi-fluorescence to reveal mRFP expression. (D-F) Recapitulated knockout phenotype. (D) Fruiting bodies of a *knkA^−^* knockout mutant developing on agar. (E) Spores of Ax2 and *knkA^−^*, stained with 0.001% Calcofluor, and imaged under phase contrast (top) and UV (bottom). (F) Spore heads of Ax2 and *knkA^−^* fruiting bodies photographed under phase contrast. Bars in A, B, D, F: 200 µm; bars in C, E: 10 µm. (G-I) Sporulation efficiency. Ax2, *knkA^−^* and 1:1 and 9:1 mixtures of Ax2 and *knkA^−^* cells were plated at 2.5 × 10^6^ cells/filter on 1 cm^2^ nitrocellulose filters supported by non-nutrient (NN) agar, and developed into fruiting bodies. (G) After vortexing the filters in 0.1% Triton X-100, the percentage of spores relative to the number of plated cells was determined. (H) The spores were clonally plated on *K. aerogenes* lawns and the percentage of emerging plaques of amoebae relative to the plated spores was determined (I). After developing the plaques into fruiting bodies, the percentage of *knkA^−^* plaques was determined. Means and SD of 3 individual experiments

To identify genes involved in *Dictyostelium* spore formation, we performed insertional mutagenesis (REMI) on Ax2 cells transformed with a fusion construct of mRFP with the spore coat gene *cotC*, expressed from its own promoter [12], and screened for mutants that lost mRFP expression. We isolated a clone, RT10_da1, that aggregated normally but formed abnormal fruiting bodies. Some aggregates terminated development by forming club-like structures that contained vacuolated cells, while others progressed into more normal looking fruiting bodies (Figure 1A). However, these structures had a relatively thick stalk that was often kinked, while the spore heads showed little CotC-RFP fluorescence (Figure 1B) and spores were not consistently elliptical (Figure 1C).

Sequencing of the genomic region flanking the inserted plasmid showed that the insertion in clone RT10_da1 occurred at a DpnII site, which was 897 nt upstream of the start codon of the nearest gene *DDB_G0271880*. To confirm that the phenotype of RT10_da1 is due to loss of *DDB_G0271880* function, we deleted part of the *DDB_G0271880* coding region (Fig. S1). The knockout cells formed more normal slugs and fruiting bodies than RT10_da1, which completed development a few hours later than wild-type cells. Similar to RT10_da1, fruiting bodies had a thick stalk that was often kinked near the tip (Figure 1D). We therefore named gene *DDB_G0271880 knkA* for *kinky(A)*. Most of the *knkA^−^* cells in the spore head were spherical and phase-dark, instead of elliptical and phase-bright like wild-type spores, and showed only weak fluorescence when stained with the cellulose dye Calcofluor (Figure 1E). The lower stalk was relatively normal with vacuolated stalk cells, but vacuolated cells spilled out of the upper stalk, which is normally encased by the stalk tube (Figure 1F). This apparent weakness of the stalk tube could be responsible for the kinked phenotype.

To assess *knkA^−^* sporulation efficiency and spore viability, and the cell-autonomous nature of the sporulation defect, we determined the percentage of detergent-resistant spores formed from known numbers of wild-type and *knkA^−^* amoebas and mixtures of the two. In contrast to wild-type cells, where the number of spores exceeds that of plated cells due to some cell division occurring after plating, only 17% of *knkA^−^* amoebas formed spores (Figure 1G) and only 40% of those germinated successfully (Figure 1H). In 1:1 wild-type/*knkA^−^* mixtures only 4% of the spores showed the *knkA^−^* phenotype after germination (Figure 1I). These data show that the *knkA^−^* mutant has a cell-autonomous defect in spore differentiation.

KnkA is a protein of unknown function, which harbors a conserved but uncharacterized UPF0183 domain. *knkA* is conserved throughout eukaryotes and is unique in their genomes. The human ortholog is annotated as C16ORF70 and is expressed in all tested cell types. The human protein atlas reports localization of C16ORF70 in nuclear speckles https://www.proteinatlas.org/ENSG00000125149-C16orf70/cell, while UniprotKB reports localization to the Golgi apparatus https://www.uniprot.org/uniprot/Q9BSU1. The methylation of *C16ORF70* is altered in some forms of schizophrenia [13], but otherwise nothing is known of the molecular or biological function of C16ORF70. The annotated homolog broad-minded http://www.ebi.ac.uk/interpro/entry/IPR005373 controls cilia formation in the mouse neural tube [14], but does not contain a recognizable UPF0183 domain and has only 14% sequence identity to mouse C16ORF70, in contrast to 33% identity of the latter with *D. discoideum* KnkA (Fig. S2A and B). A phylogenetic tree, annotated with domain architectures and for *Dictyostelia* expression profiles shows that all KnkA orthologs have the UPF0183 domain, which is sometimes interrupted by low complexity sequence. *knkA* transcripts increase gradually upon starvation in taxon-group representative *Dictyostelia* and are about equally expressed in pre-stalk and pre-spore cells, but show preferential expression in the stalk of mature fruiting bodies (Fig. S2C).

### Further characterization of the knka^−^ phenotype

#### Cell type-specific gene expression

**Figure 2.**
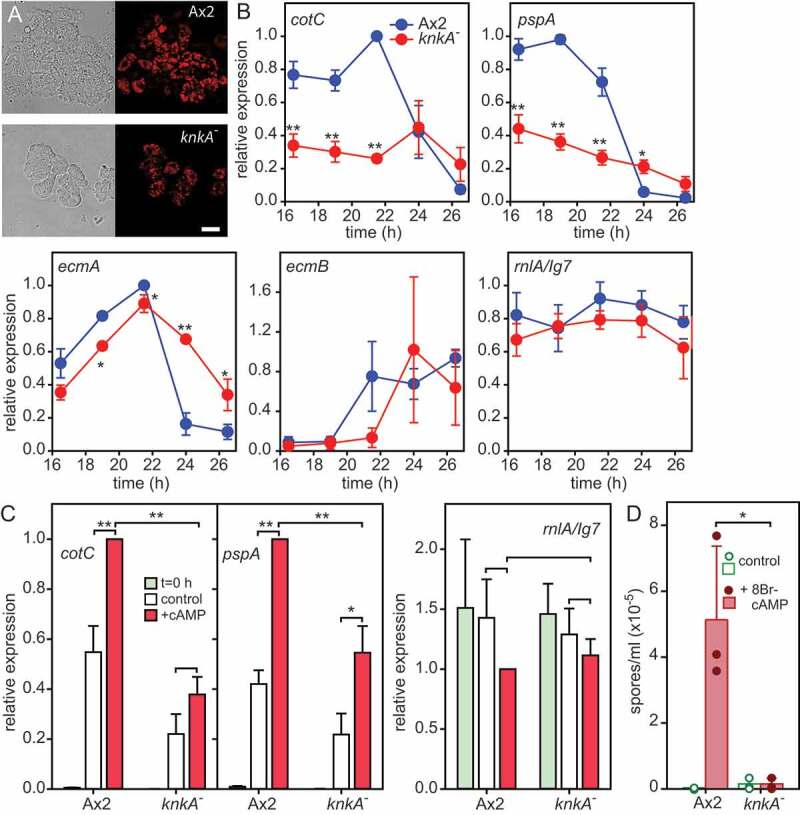
Cell differentiation and cell type-specific gene expression in *knkA^−^*. (A) Pre-spore vesicles in *knkA*^−^ slugs. Ax2 and *knkA^−^* cells were transformed with CotC-mRFP expressed from the *cotC* promoter and developed into migrating slugs, which were then dissociated and imaged by confocal microscopy. Left panels: transmitted light, right panels mRFP fluorescence. Bar: 10 µm. (B) Cell-type specific gene expression. Developing Ax2 and *knkA^−^* structures were harvested at different time points between slug formation (16 h) and completion of fruiting body formation (26 h) for Ax2. Total RNA was isolated and probed by qRT-PCR with primers specific to the pre-spore genes *pspA* and *cotC*, the pre-stalk genes *ecmA* and *ecmB* and the constitutively expressed gene *rnlA/Ig7*. Means and SD of 3 experiments assayed in duplicate are presented. (C) cAMP induction of pre-spore gene expression. Ax2 and *knkA^−^* cells were developed into loose aggregates, which were dissociated and incubated at 3 × 10^6^ cells/ml in the presence and absence of 1 mM cAMP. After 0 and 4 h of incubation total RNA was isolated and probed by RT-qPCR with *cotC-, pspA-* and *rnlA-*specific primers. Means and SD of 3 experiments assayed in duplicate are presented. (D) 8Br-cAMP induction of sporulation. Cells from dissociated Ax2 and *knkA^−^* slugs were shaken at 10^6^ cells/ml in KK2 with 1 mM MgCl_2_ in the presence and absence of 10 mM 8Br-cAMP. After 8–10 h, the density of spore cells was determined. Individual data, means and SD from 3 experiments are presented. Differences between Ax2 and *knkA^−^* values at the same time point (B) and between control and cAMP (C) or 8Br-cAMP (D) treated samples were tested for significance using a t-test or rank-sum test when data were not normally distributed *:P < 0.05; **:P < 0.005

Following aggregation, the *Dictyostelium* spore coat is partially pre-fabricated on the interior face of Golgi-derived pre-spore vesicles that fuse with the plasma membrane at the onset of sporulation [15]. Many spore-coat genes, such as *cotC* are expressed at this stage, in response to increasing cAMP levels [11,16]. We examined dissociated slugs of Ax2 and *knkA^−^* cells transformed with CotC-mRFP to assess the formation of pre-spore vesicles. Figure 2A shows that Ax2 cells contained many RFP-positive vesicles and that similar vesicles were also present in *knkA^−^*, indicating that initial pre-spore differentiation was not very defective. To quantify the expression of a range of cell type-specific genes, we collected total RNAs of developing Ax2 and *knkA^−^* mutants from the early slug-stage onward and measured levels of pre-spore and pre-stalk transcripts by RT-qPCR. Transcripts of the pre-spore genes *cotC* and *pspA* were about 60% reduced in *knkA^−^* development, while those of the pre-stalk gene *ecmA* and stalk gene *ecmB* were not markedly different, although *ecmA* decreases and *ecmB* increases about 2 h later. *ecmB* is only well expressed in culmination, which is not captured here for *knkA^−^* cells, due to their delayed development. We therefore also compared transcripts for *ecmB* and the spore gene *spiA* between equivalent developmental stages of Ax2 and *knkA^−^*, rather than time points (Fig. S3). Expression of the spore gene *spiA* was about 75% reduced in *knkA^−^*, while both *ecmB* and the constitutively expressed gene *rnl7*/*ig7* were 25-30% reduced. Overall, it appeared that pre-spore and spore gene expression showed a marked decrease in *knkA^−^*, while pre-stalk genes were relatively unaffected. Pre-spore gene induction in early slugs requires extracellular cAMP acting on cAMP receptors [16,17], and for several genes like *cotC* also intracellular cAMP acting on PKA [18]. We compared cAMP induction of pre-spore gene expression between Ax2 and *knkA^−^* cells and found that absolute levels of expression of both the PKA-independent gene *pspA* and the PKA-dependent gene *cotC* were respectively, 40% and 50%, reduced in *knkA^−^* cells (Figure 2C), but that the *knkA^−^* cells still showed some response to cAMP.

In wild-type cells from dissociated slugs, spore formation can be induced by incubation with the membrane-permeant PKA activator 8Br-cAMP, which at the high concentrations required for PKA activation will also activate cAMP receptors [17]. Figure 2D shows that 8Br-cAMP induced from 37% to 77% of Ax2 cells to differentiate into spores in suspension, but this was less than 8% for *knkA^−^* cells. Evidently, PKA activation cannot bypass the sporulation defect of *knkA^−^.*

#### Slug migration

**Figure 3.**
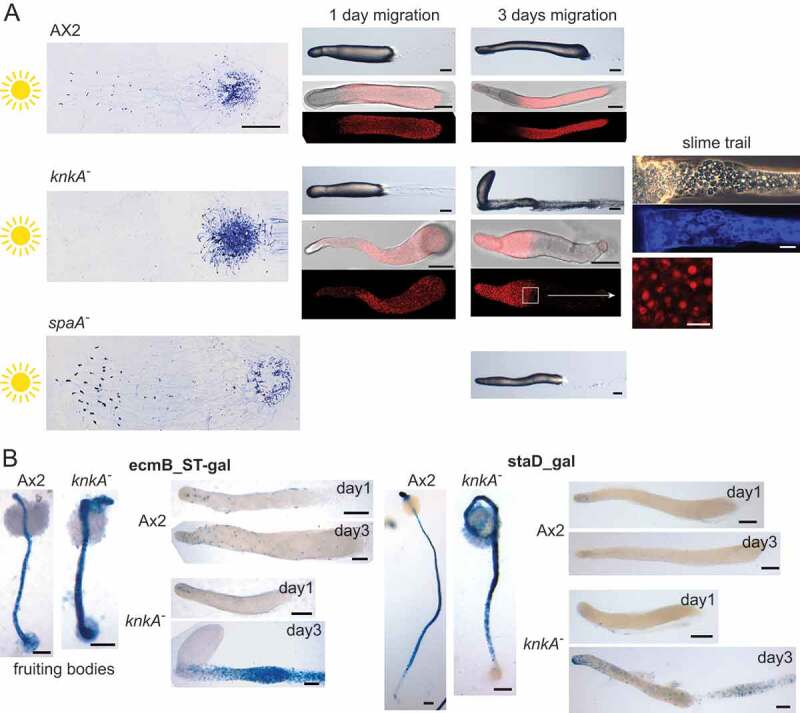
Slug migration and phototaxis. (A) Phototaxis. Ax2 and *knkA^−^* cells, both untransformed and transformed with CotC-mRFP, as well as *spaA^−^* cells were placed under unilateral light for 3 d. Slugs were then lifted from the agar by overlay with a piece of overhead sheet and stained with Coomassie Blue to visualize the slugs and slime trails (left panels, bar: 1 cm). After 1 and 3 d CotC-mRFP transformed slugs were examined by confocal microscopy (bars: 100 µm and 10 µM for inset) and the slime trail of a 3-d-old *knkA^−^* slug was stained with Calcofluor (middle right panel, bar: 10 µm). The experiment was repeated once with similar results. (B) Stalk gene expression. Ax2 and *knkA^−^* cells, transformed with *ecmB_ST-gal* or *staD-gal*, were set up for normal development into fruiting bodies and for phototaxis as above. Fruiting bodies and slugs that had migrated for 1 or 3 d were fixed and stained with X-gal. Bars: 100 µm

Under ambient light, *knkA^−^* slugs directly form fruiting bodies, but they can be induced to migrate when exposed to unilateral light. (Figure 3A). Control Ax2 slugs migrated several centimeters toward light before turning into fruiting bodies. However, while the *knkA^−^* slugs initially showed some phototaxis, they then left most cells behind in the slime trail and stopped migrating. The discarded cells were highly vacuolated and encased in cellulose, similar to wild-type stalk or basal disc cells. *knkA^−^* cells transformed with CotC-mRFP initially showed normal CotC expression in the slug posterior. CotC-mRFP then became localized into a single large vacuole before being lost altogether. The remaining cells formed a club-like structure, where cells remained amoeboid and never formed a fruiting body. To test whether the migration phenotype is related to the poor expression of pre-spore genes in *knkA^−^* cells, we also tested slug migration in *spaA^−^*, which has much-reduced spore coat gene expression [12]. *spaA^−^* normally migrated, suggesting that defective pre-spore gene expression and defective migration are 2 separate consequences of loss of *knkA*.

To understand the apparent trans-differentiation of *knkA^−^* cells during slug migration, we transformed *knkA^−^* and wild-type cells with fusion constructs of the *LacZ* reporter gene (gal) and the *ecmB_ST* promoter [19], which is expressed in both the stalk and basal disc of fruiting bodies, and the *staD* promoter, which is only expressed in the stalk [20]. Figure 3B shows that like Ax2, *knkA^−^* fruiting bodies expressed *ecmB_ST* in the stalk and the basal disc, while *staD* was only expressed in the stalk. However, conform to its overall phenotype (Figure 1C), the *knkA^−^* stalk was both thicker and kinked at the top. In early- and late-migrating Ax2 slugs, *ecmB_ST* was expressed at the tip core and in some cells scattered throughout, but in late migrating *knkA^−^* slugs, *ecmB-ST* expression was very strong in the slug trail. This was not the case for *staD*, which was expressed somewhat scattered throughout the slug and trail. This result suggests that the vacuolated cells in the *knkA^−^* slug trail were not stalk cells, but basal disc cells.

### Identification of Bcas3 as a protein that interacts with Knka

**Figure 4.**
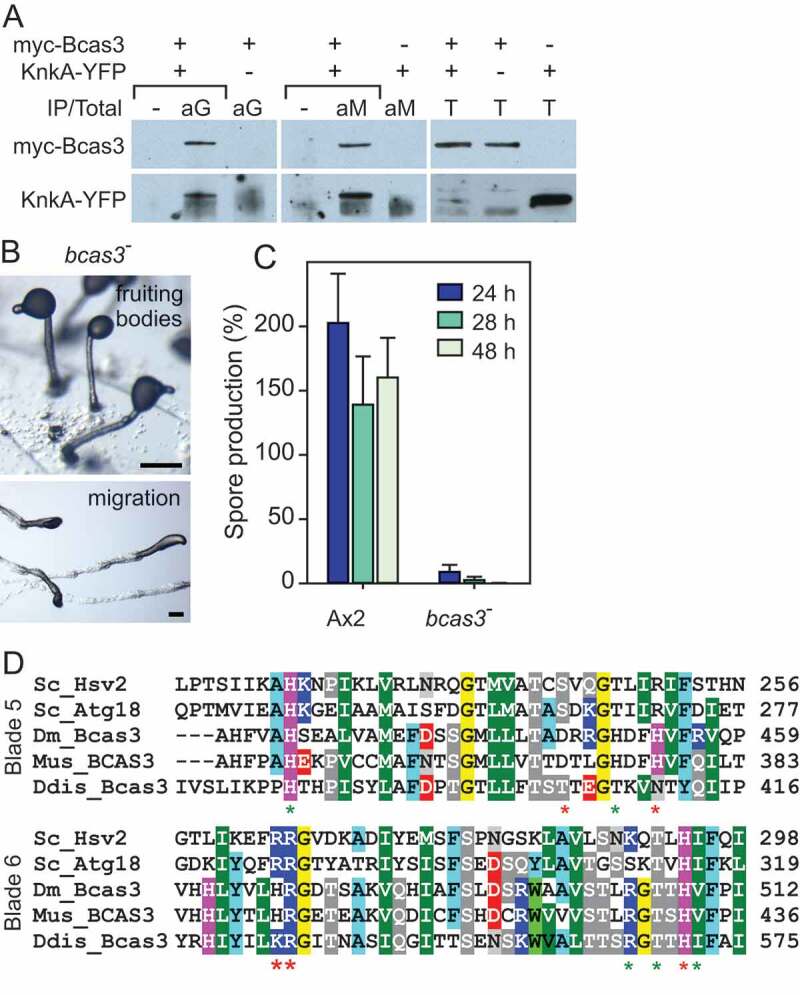
Bcas3/KnkA interaction, *bcas3^−^* phenotype, and putative PtdinsP binding. (A) Co-immunoprecipitation. *knkA^−^* cells were transformed with either KnkA-YFP or MYC-Bcas3 constructs or with both together. Slug cell lysates were immuno-precipitated with either anti-GFP (aG) or anti-MYC (aM) antibodies as indicated, and both the immunoprecipitates and total lysates (T) at 1/70^th^ of the amount used for immunoprecipitation were size-fractionated on SDS-PAG(E). Western blots were probed with the anti-MYC (top row) and anti-GFP (bottom row) antibodies. (B) *bcas3^−^* phenotype. *bcas3^−^* cells were developed into fruiting bodies (top) or set up for slug migration under lateral light (bottom). Bar: 200 µm (C) Spore production. *bcas3^−^* cells were plated at 2.5 × 10^6^ cells/filter on 1 cm^2^ nitrocellulose filters and developed into fruiting bodies for the indicated time periods. After vortexing the filters in 0.1% Triton-X100, the percentage of spores relative to the number of plated cells was determined. Means and SD of 3 experiments. (D) Alignment of *D. discoideum* Bcas3 with the PtdIns3P binding regions of yeast Hsv2 and Atg18. Red or green asterisks mark amino-acids with essential or supporting roles in PtdIns3P binding, respectively [3,28]

To gain insight into the molecular function of KnkA, we used immunoprecipitation with anti-GFP antibodies to identify proteins that interact with KnkA-YFP. *knkA^−^* cells that overexpress KnkA-YFP from the constitutive *act15* promoter were developed to early culminants, lysed and cross-linked with 1 mM di(N-succinimidyl) 3,3ʹ-dithiodipropionate (DSP). The lysate was immunoprecipitated with GFP-trap agarose and bound material was extracted with SDS buffer and size-fractionated (Fig. S4A). Gel segments of the cross-linked proteins from *knkA^−^* and *knkA^−^*/A15-KnkA-YFP cells were subjected to LC-MS-MS mass-spectrometry. A single protein, DDB_G0272949, was significantly enriched in 3 experiments (Fig. S4B, Table S2). *DDB_G0272949* is homologous to *BCAS3*, a gene upregulated in breast cancer and other malignancies [21,22], which is essential for angiogenesis and survival of mouse embryos [23], where BCAS3 localizes to microtubules and intermediate filaments [24,25]. To confirm the interaction of *Dictyostelium* Bcas3 with KnkA, we co-expressed MYC-tagged Bcas3 with KnkA-YFP in *knkA^−^* cells, performed immunoprecipitation with anti-MYC antibodies and probed the size-fractionated precipitate with GFP antibodies, or conversely immunoprecipitated with anti-GFP antibodies and probed the precipitate with anti-MYC antibodies (Figure 4A). The first experiment showed specific pull-down of KnkA-YFP by Bcas3 and the second pull-down of Bcas3-MYC by KnkA, confirming the affinity of the 2 proteins for each other.

Phylogenetic analysis of *D. discoideum* Bcas3 and its closest relatives in a range of eukaryote phyla showed that *D. discoideum* Bcas3 belongs to the same clade as the metazoan Bcas3 orthologs, but is somewhat more closely related to a set of plant proteins computationally annotated as Atg18, however without experimental support for a role of these proteins in autophagy (Fig. S5). The well-characterized yeast Atg18 grouped with *D. discoideum* Atg18, other plant Atg18s and with the metazoan Atg18 homologs that are known as WIPI proteins (WD repeat domain, phosphoinositide interacting), which perform a scaffold function in autophagy [26]. As a group, they clustered more closely with other WD40 repeat (wdr) proteins, such as WDR45 and yeast Hsv2. Similar to WDR45 and Hsv2, the Atg18 proteins consist mostly of 7 WD40 repeats, which are protein-protein interaction domains that assume a 7-bladed beta-propeller fold [27]. WD40 repeats are also present in the Bcas3 proteins, but are then followed by the Bcas3 domain. Overall, the Bcas3 proteins are also 3 times larger than the Atg18 and WDR45 proteins. All 3 types of proteins were, like KnkA, not detected in prokaryotes, but are present across eukaryotes, indicating that they are part of the core gene repertoire of eukaryotes. The dictyostelid *bcas3* genes are like the *knkA* genes upregulated during development, *bcas3* is somewhat higher expressed in pre-spore than pre-stalk cells, but like *knkA* shows in *D. discoideum* highest expression in stalk cells (Fig. S5).

To identify the function of *D. discoideum bcas3*, we generated a *bcas3* knockout (Fig. S1B). The phenotype of the *bcas3^−^* cells was very similar to that of *knkA^−^*. They also formed fruiting bodies with short, thick, and kinked stalks and abnormal spores with poor viability (Figure 4B). When induced to migrate, slugs left large amounts of cells behind in their trail and never formed fruiting bodies. This result strongly suggests that Bcas3 and KnkA not only interact but work together to perform the same cellular function.

### Bcas3 is a likely inositol phospholipid-binding protein

Both Hsv2, Atg18 and the WIPI proteins contain binding sites for PtdIns3P or PtdIns(3,5)P_2_ across 2 of their WD40 repeats [3,28,29]. These binding sites are essential for the recruitment of Atg18 to autophagosome membranes, where Atg18 causes membrane scission and vesicle fission into smaller compartments [9].

**Figure 5.**
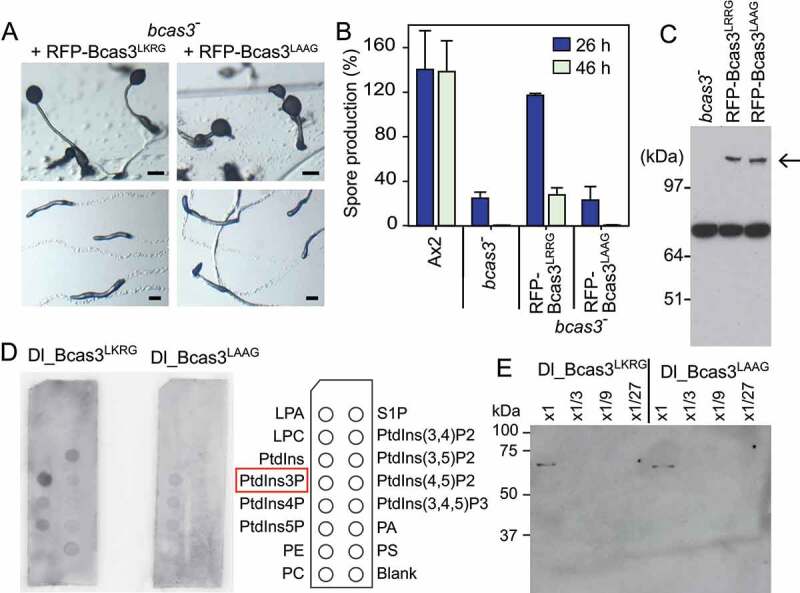
Effects of mutation of the PtdIns3P binding site of Bcas3. (**A/B**) Mutation of the LKRG motif. *bcas3^−^* cells were transformed with either wild-type RFP-Bcas3 or a construct in which the LKRG essential for PtdIns3P binding was mutated to LAAG. Transformants were developed under ambient or unilateral light and photographed (A), or assayed for spore production (B) as described for panel 4C. Bar: 200 µm. (C) Western blot of lysates of *bcas3^−^* and *bcas3^−^*, transformed with RFP-Bcas3^LKRG^ or RFP-Bcas3^LAAG^ and probed with anti-GFP antibodies. (D) Phospholipid binding activity. His-Dl-Bcas3^LKRG^ and His-Dl-Bcas3^LAAG^ were expressed and purified from *E.coli*. About 200 ng protein was exposed to PIP strips with 15 different phospholipids and visualized with HRP-conjugated anti-His antibodies. Representative image of 3 experiments. (E) Western blot of serial dilutions of the His-Dl-Bcas3^LKRG^ and His-Dl-Bcas3^LAAG^ protein preparations used in (D), probed with anti-His antibodies to compare protein concentrations. The undiluted sample was used

Most residues involved in PtdIns3P and PtdIns(3,5)P_2_ binding are well conserved between Hsv2/Atg18/WIPI and *D. discoideum* Bcas3 (Figure 4D). Particularly, the 2 basic pockets created by RR residues in the FRRG motif (LKRG in *D. discoideum* Bcas3) are essential for binding to inositol phospholipids. To assess whether inositol phospholipid-binding is also essential for the role of *D. discoideum* Bcas3, we mutated its LKRG motif to LAAG and expressed both Bcas3^LKRG^ (wild type) and the Bcas3^LAAG^ proteins fused to RFP in the *bcas3^−^* mutant (Figure 5C). The Bcas3^LKRG^-RFP protein almost completely restored slug migration, fruiting body morphology, and spore viability in the *bcas3^−^* mutant (Figure 5A and B), although the spores were not completely stable. However, Bcas3^LAAG^-RFP transformed *bcas3-* cells retained the same defects as *bcas3^−^*. It is unclear why the Bcas3^LKRG^-RFP protein did not fully restore development; possibly, the attached RFP interferes somewhat with protein function, or there is a gene dosage effect. However, the experiment suggested that inositol phospholipid-binding is essential for the function of Bcas3.

To directly assess the phosphoinositide-binding activity of Bcas3 and Bcas3^LAAG^, we first attempted to express and purify both *D. discoideum* proteins from *E. coli* or *D. discoideum*, but the yield was very low. The poor expression in *E.coli* was likely due to the high content of low complexity A/T rich sequence in *D. discoideum bcas3*. The compact *Dictyostelium lacteum* genome mostly lacks such sequence [30], while otherwise, the unique *D. lacteum* Bcas3 protein (DLA_09359) shares high sequence similarity with the unique *D. discoideum* Bcas3 (Fig. S5). His-tagged *D. lacteum* Bcas3 and Bcas3^LAAG^ proteins could be expressed to low but sufficient levels in *E. coli* (Figure 5E). Wild-type Bcas3 predominantly bound to PtdIns3P and somewhat to PtdIns(3,5)P_2_ and PtdIns5P on membrane strips containing 15 different phospholipids, while Bcas3^LAAG^ showed almost no phospholipid binding activity (Figure 5D). PtdIns3P and PtdIns3,5P_2_ are also the targets for other proppins [3], making it very likely that Bcas3 is a proppin.

### Localization of Knka and Bcas3

**Figure 6.**
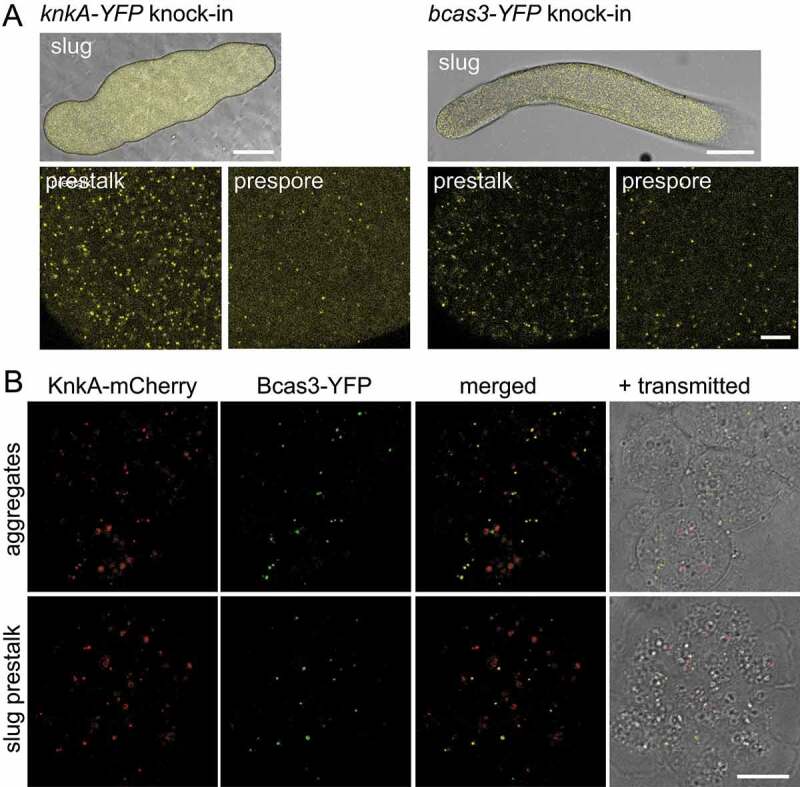
KnkA and Bcas3 colocalization in structures and single cells. (A) Intact slugs. Ax2 cells that harbored a YFP knock-in in either their *knkA* or *bcas3* genes were developed into slugs and imaged by confocal microscopy at low magnification (top, bars: 100 µm), or higher magnification (bars: 10 µm) separately in the pre-stalk and pre-spore regions. (B) Single cells. Ax2 cells harboring both a *mCherry* knock-in in *knkA* and a YFP knock-in in *bcas3* were developed to aggregates and slugs. Dissected slug pre-stalk regions and aggregates were mechanically dissociated and imaged by confocal microscopy. Individual and merged images are shown, the latter also merged with the transmitted light image (bar: 10 µm)

To investigate KnkA and Bcas3 localization in cells and structures, we knocked-in YFP tags at the 3ʹ ends of the *knkA* and *bcas3* genes (Fig. S1C and E) of Ax2. KnkA-YFP and Bcas3-YFP were expressed in both the pre-stalk and pre-spore region of slugs, with expression in pre-stalk cells showing a more punctate distribution than in pre-spore cells for particularly KnkA-YFP (Figure 6A). To show possible colocalization of KnkA and Bcas3, we also generated a double knock-in of mCherry in *knkA* and YFP in *bcas3* (Fig. S1E). Figure 6B shows that apart from some diffuse larger patches, all KnkA-mCherry puncta colocalized with YFP-Bcas3 puncta in cells from both dissociated aggregates and the dissociated pre-stalk region of slugs. Individual cells from the pre-spore region contained too few puncta to demonstrate this in a single image. Cells from aggregates adhered better to glass slides and were preferentially used in further studies, with pre-stalk cells shown in supplemental data.

**Figure 7.**
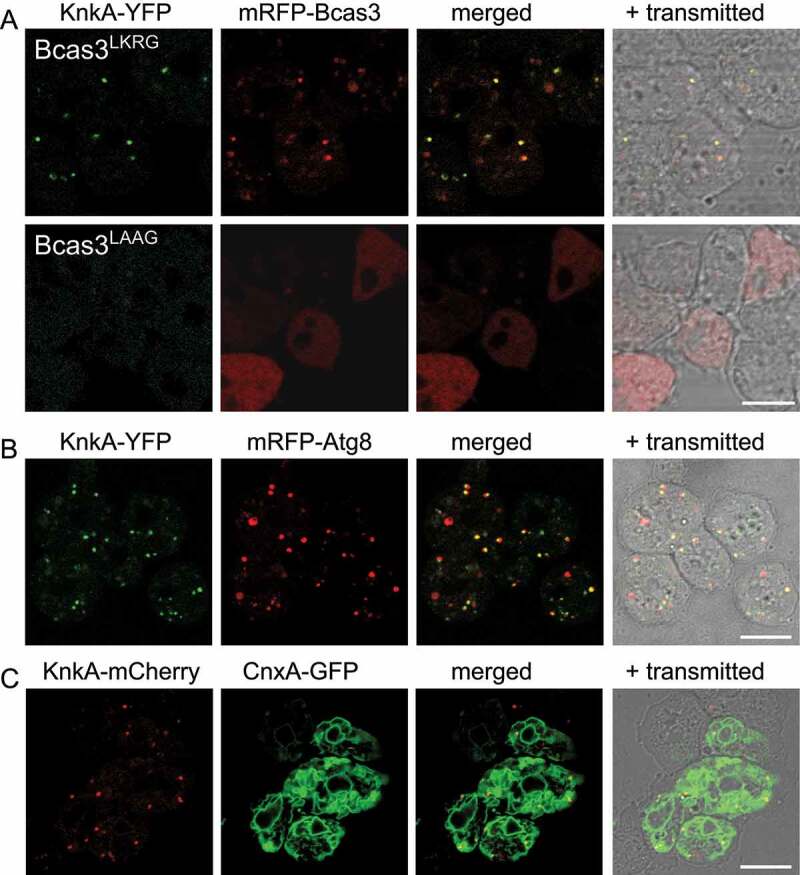
KnkA recruitment by Bcas3 and colocalization with Atg8 and CnxA. (A) KnkA recruitment. Dissociated aggregates of *bcas3^−^*/*knkA-YFP* knock-in cells, transformed with [*bcas3*]-*mRFP-bcas3*^LKRG^ (wild-type) or [*bcas3*]-*mRFP-bcas3*^LAAG^ were imaged by confocal microscopy. Individual and merged images are presented as in figure 6B. (B) KnkA and Atg8 localization. Dissociated aggregates of Ax2 *knkA-YFP* knock-in cells transformed with *mRFP-atg8* were imaged, as in 6B. (C) KnkA and CnxA. Dissociated aggregates of Ax2 *knkA-mCherry* knock-in cells, transformed with *cnxA-GFP*, were imaged as in 6B, bars:10 µm

We next investigated whether the punctate distributions of KnkA and Bcas3 were dependent on the phospholipid-binding domain of Bcas3. *bcas3^−^* cells, harboring a KnkA-YFP knock-in and further transformed with either wild-type (LKRG) [*bcas3*]-*mRFP-bcas3* or [*bcas3*]-*mRFP-bcas3* harboring the LAAG mutation in its phospholipid-binding domain were developed into aggregates and slugs, and we imaged both dissociated aggregates (Figure 7A) and slug pre-stalk regions (Fig. S6A) for KnkA-YFP and mRFP-Bcas3 fluorescence. While *bcas3^−^* cells transformed with Bcas3^LKRG^ showed both punctate distribution of KnkA and Bcas3, which was mostly colocalized, punctate KnkA and Bcas3 distribution was lost from the *bcas3^−^*/Bcas3^LAAG^ cells. These results show that both KnkA and Bcas3 localization to puncta are dependent on an intact Bcas3 phospholipid-binding domain.

To identify the nature of the KnkA and Bcas3 puncta, we performed colocalization of KnkA-mCherry with the endoplasmic reticulum (ER) marker CnxA (calnexin) [31], the Golgi marker Gol (golvesin) [32], the contractile vacuole marker GppA (dajumin) [33], all fused to GFP, and the peroxisome targeting peptide SKL fused to YFP [34], and of KnkA-YFP with the autophagosome marker RFP-Atg8 [35] and the lysosome marker LysoTracker Red. The KnkA puncta did not colocalize with Gol, GppA, SKL peptide or LysoTracker Red (Fig. S6B). However, they mostly coincided with the large domain outlined by the ER marker CnxA (Figure 7C) and with the smaller vesicles that expressed the autophagosome marker mRFP-Atg8 in dissociated aggregates (Figure 7B). Strikingly, the KnkA-YFP puncta were always localized off-center from the mRFP-Atg8 vesicles. In dissociated slug pre-stalk cells, mRFP-Atg8 was mostly localized to relatively large vesicles and colocalization with KnkA-YFP was rare (Fig. S6B). These data suggest that KnkA-YFP colocalizes with early autophagosomes. However, its absence from larger late and acidified autophagosomes could also be due to the pH sensitivity of YFP. The observed colocalization of KnkA-YFP with CnxA is consistent with its position at the PAS, which is localized at the ER in mammals [36] and *Dictyostelium* [37].

### Autophagy is impaired in knka^−^ and bcas3^−^ cells

The above results suggest that KnkA is recruited to the PAS or early autophagosomes by Bcas3 via its phospholipid-binding activity. We analyzed if autophagy is affected in *knkA^−^* and *bcas3^−^* cells using RFP-GFP-Atg8 [38]. RFP-GFP-Atg8 marks early autophagosomes of neutral pH yellow, due to the combined RFP and GFP fluorescence, but mature autolysosomes become red due to quenching of GFP fluorescence by low pH.

**Figure 8.**
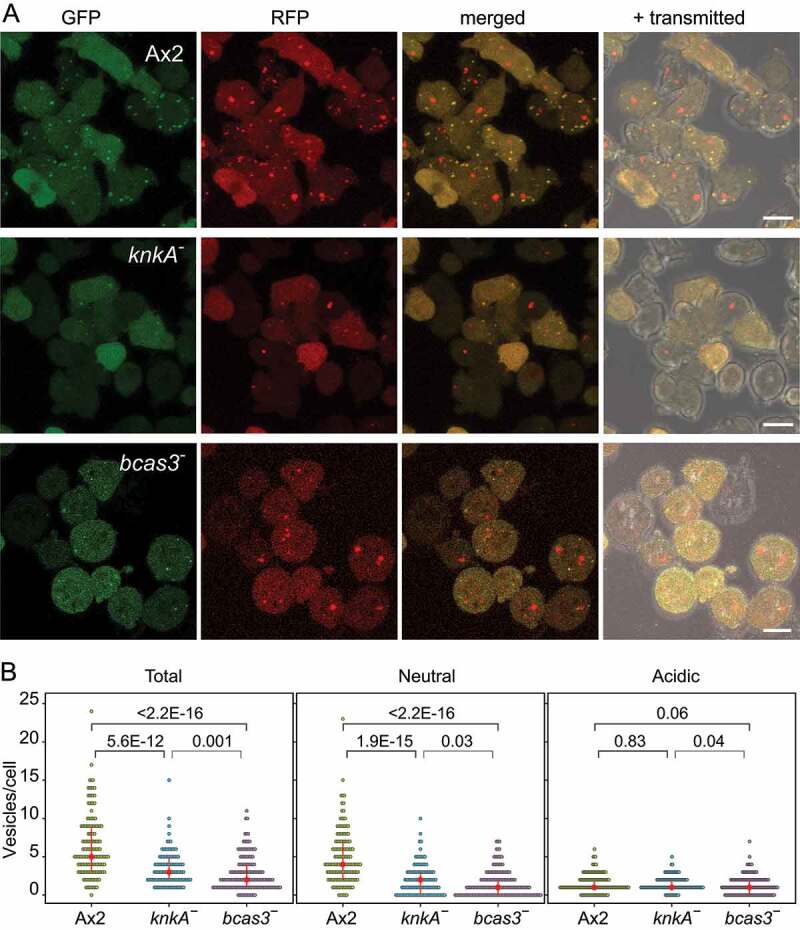
Autophagy in *knkA-* cells. (A) Microscopy. Dissociated aggregates of Ax2, *knkA^−^* and *bcas3^−^* cells, transformed with RFP-GFP-Atg8, were imaged by confocal microscopy. Z series of images in 0.3–0.5 µm steps were captured and the maximum intensity projection of these images, as shown here, was used to quantify Atg8 vesicles (bar: 10 µm). (B) Quantification. Numbers of vesicles in each cell that showed both RFP and GFP fluorescence (neutral), or only RFP fluorescence (acidic) were counted, and the sum of neutral and acidic vesicles (total) was calculated. Red circles and red bars represent the median and quartile of each data set, respectively. The numbers above the brackets are P-values of significant differences between Ax2, *knkA^−^* and *bcas3^−^* cells (Wilcoxon rank-sum test), n = 149 for Ax2, 118 for *knkA^−^* and 157 for *bcas3.*

Aggregating cells of Ax2 expressing RFP-GFP-Atg8 showed many small yellow vesicles as well as some larger red vesicles. In contrast, *knkA^−^*/RFP-GFP-Atg8 and *bcas3^−^*/RFP-GFP-Atg8 cells showed some large red vesicles, but much fewer yellow ones (Figure 8 and S7A). Quantification of Atg8-positive vesicles showed that the total number of Atg8-positive vesicles in Ax2 cells was about 2-fold higher than in *knkA^−^* or *bcas3^−^* cells, with over 3-fold as opposed to ~ 1.2-fold more neutral early phagosomes than acidic mature phagosomes. (Figure 8B, Table S3). In slugs, Ax2 pre-stalk cells have more mature acidic vesicles, while *knkA^−^* and *bcas3^−^* pre-stalk cells have overall much less autophagic vesicles (Fig. S8A, C, and Table S3) and this is more strongly the case for pre-spore cells (Fig. S8B and C). Overall, the results indicate that autophagic activity is impaired in *knkA^−^* and *bcas3^−^* cells.

## Discussion

### The phenotype of the knka^−^ mutant is complex

*knkA* was identified as the defective gene in a REMI mutant with a cell-autonomous defect in spore production. However, both the REMI mutant and a *knkA^−^* knockout generated by homologous recombination also showed other defects. Stalks failed to reach their proper length and thinly tapered morphology. They showed a kink in their upper part and expulsed cells from their top when placed under slight pressure (Figure 1), which could be a consequence of the stalk tube being relatively weak. The sporulation defect of *knkA^−^* was accompanied by reduced expression and cAMP-inducibility of pre-spore genes, while the expression of pre-stalk and stalk genes was relatively normal (Figure 2). Under unilateral light, which favors slug migration, *knkA^−^* slugs initially migrated, but then halted with pre-spore cells progressively de-differentiating into vacuolated basal disc cells, which were lost from the rear (Figure 3). Another sporulation-defective mutant, *spaA^−^*, showed normal slug migration, indicating that defective migration and excessive basal disc differentiation are additional consequences of loss of *knkA*.

### Knka interacts with the proppin Bcas3, a phosphoinositide-binding protein

Immunoprecipitation of slug lysates with KnkA-YFP, followed by mass spectrometry, identified a *D. discoideum* homolog of mammalian BCAS3 with significant statistical support (Fig. S4) as a KnkA-binding protein. Deletion of *D. discoideum bcas3* yielded a mutant with the same spore, stalk and slug migration-defective phenotype as *knkA^−^* (Figure 4), indicating that Bcas3 and KnkA are involved in the same biological process. While KnkA only contains a conserved domain of unknown function, Bcas3 shows homology with proppins (beta-propellers that bind phosphoinositides) and specifically bound to the proppin target PtdIns3P on strips spotted with phosphoinositides and other lipids (Figure 5). The ability to bind to PtdIns3P was lost in mutant Bcas3, where 2 basic amino-acid residues in the LKRG motif, which are essential for phosphoinositide binding in well-characterized proppins, were mutated to LAAG. In wild-type slugs and aggregates, KnkA and Bcas3 colocalized to puncta (Figure 6), but this localization was also lost in cells harboring Bcas3^LAAG^ (Figure 7). In contrast to wild-type Bcas3, Bcas3^LAAG^ also did not restore normal migration and sporulation in the *bcas3^−^* mutant (Figure 4), indicating that the phosphoinositide binding domain of Bcas3 is essential for its function.

### Knka colocalizes with Atg8 at the PAS and is required for autophagy

Co-expression of KnkA-YFP or KnkA-mCherry with a range of RFP or GFP tagged organelle markers showed that KnkA was not localized at the Golgi, in lysosomes or peroxisomes or contractile vacuoles (Fig. S6). KnkA puncta mostly localized to the extensive ER network and colocalized off-center to smaller but not larger vesicles that contain Atg8 (Figure 7). This localization associates KnkA with the PAS, the nucleation site for double membranes that will envelop cellular materials for autophagy. The ER acts as a platform for recruitment of many autophagy proteins, amongst which the PtdIns3K kinase complex, which generates PtdIns3P binding sites for recruitment of the proppin Atg18/WIPI, and Atg8/LC3, which becomes tethered to phosphatidylethanolamine in the phagophore membrane and contributes to phagophore expansion and fusion with lysosomes [39]. The association of KnkA and Bcas3 with autophagy was further confirmed by the observation that starving *knkA^−^* and *bcas3^−^* cells show greatly reduced numbers of Atg8 containing vesicles, compared to wild-type cells (Figure 8, S7, and S8).

### knka^−^ and bcas3^−^ mutants resemble atg7^−^, atg5^−^ and atg9^−^ mutants

Our mutagenesis approach recently identified *atg7* as another gene required for pre-spore gene expression [11]. The *atg7^−^* phenotype was more severe than that of *knkA^−^* or *bcas3^−^*. Pre-spore gene expression and its induction by cAMP were almost completely lost, and the “pre-spore” population trans-differentiated into vacuolated stalk-like cells. Aggregates gave rise to multiple tips developing into sorogens, which could not be induced to migrate at all. The sorogens formed misshapen fruiting bodies after a long delay. Mutants in *atg5^−^* and *atg9^−^* showed the same phenotype with almost complete lack of cAMP induction of pre-spore gene expression, trans-differentiation of pre-spore into stalk-like cells [11] and the multi-tipped aggregates that are also characteristic of many other *Dictyostelium* mutants in autophagy genes, such as *atg8, tipD/atg16, atg101* and *tipC/vps13* [40–43]. In *knkA^−^* and *bcas3^−^* mutants pre-spore gene expression was only ~60% reduced and pre-spore cells only trans-differentiated into stalk-like cells during prolonged slug migration. There was no obvious multi-tipped phenotype and fruiting body formation was much less delayed. However, the spectrum of changes was similar and the data overall suggest that KnkA and Bcas3 are also required for autophagy, but that their loss still allows some autophagy to take place.

### Knka and Bcas3 possibly also interact in mammals and Bcas3 functions in mouse angiogenesis

Similar to *knkA, bcas3* is deeply conserved in eukaryotes, usually as a single copy gene, and was first identified as a gene upregulated in breast cancer and other malignancies [21,22]. The N-terminal region of Bcas3 contains its lipid-binding proppin region, but Bcas3 is larger than other proppins and contains an additional Bcas3 domain. The protein-protein interaction database STRING [44] associates human Bcas3 with the human KnkA ortholog C16ORF70 and *vice versa* based on co-expression data https://string-db.org/cgi/network.pl?taskId=K9XvOtCA5kpN, but a physical interaction between the 2 proteins has to our knowledge not been reported.

During mouse embryonic development, BCAS3/Rudhira, the mouse ortholog of Bcas3, is expressed in angiogenic precursors [45] and colocalizes with microtubules and intermediate filaments [24]. During wound healing of endothelial cells, BCAS3-positive filaments become more prominent at the leading edge of the cell, where BCAS3 recruits and activates CDC42 and facilitates actin reorganization. *bcas3* knockdown prevents cell migration and wound healing [24], while *bcas3* knockout mice are embryonic lethal with severe defects in angiogenesis [23]. The BCAS3 domain is both necessary and sufficient for its binding to TUB/tubulin and VIM (vimentin) intermediate filaments and its role in cell migration, while its WD40 repeats have no obvious role [25].

Both the localization and molecular function of mouse BCAS3 are quite different from those of *Dictyostelium* Bcas3, where no association with microtubules was evident from cellular localization studies and where the WD40 repeat 6 with its phosphatidylinositol binding motif was essential for its function (although a separate role for the Bcas3 domain was not investigated). Considering the vast evolutionary distance between *Dictyostelium* and mammals, it is possible that the roles of Bcas3 in either lineage have greatly diverged, but it would be worthwhile to investigate in either *Dictyostelia* or metazoa whether vestiges of alternative Bcas3/BCAS3 functions are not present.

### Future prospects

Our analysis of *D. discoideum* sporulation-deficient mutants highlighted known and novel autophagy genes with crucial roles in sporulation and may continue to yield novel components of the autophagy machinery. The deep conservation of both Bcas3 and KnkA in eukaryotes suggests that their roles in autophagy are likely to extend beyond *Dictyostelia*. Much is yet to be learned of the molecular functions of KnkA and Bcas3 and their role in early phagosome assembly. The excellent amenability of *Dictyostelium* for both genetic and cell biological approaches and its dependence on autophagy to proceed through its starvation-induced life cycle makes the organism particularly suitable for investigating autophagy.

## Materials and methods

### Cell culture

*Dictyostelium discoideum* Ax2 was cultured either in HL5 axenic medium (Formedium, HLG0102) or on SM agar plates (Formedium, SMAA0102) in association with *Klebsiella aerogenes*. For development, cells were distributed at 2.5 × 10^6^ cells/cm^2^ on non-nutrient agar (1.5% Bacto-agar [BD Biosciences, 214010] in 8.8 mM KH_2_PO_4_ and 2.7 mM Na_2_HPO_4_) and for beta-galactosidase staining on dialysis membrane (BDH, 275127004) supported by non-nutrient agar. Visualization of beta-galactosidase expression in intact structures was performed using established procedures [46]. To analyze slug migration, cells were either streaked or spotted at 2.5 × 10^5^ cells/cm^2^ on 1.5% agar (BD Biosciences, 214010) in water and incubated under unidirectional light for 3 d. The slugs plus slime trails were lifted onto a plastic sheet and stained with Coomassie Brilliant Blue.

### Plasmid construction and transformation

All oligonucleotide primers used in this work are listed in **Table S1**. Plasmids are constructed as follows: *knkA* knockout vector: 5ʹ and 3ʹ fragments of the *knkA* genomic region (**Fig. S1A**) were amplified using primer pairs *knkA-f1/knkA-r1* and *knkA-f2/knkA-r2,* respectively, and sub-cloned into the pJet1.2/blunt vector (ThermoFisher, K1231). The fragments were excised and successively inserted into plasmid pLPBLP [47] using HindIII/SalI for the 5ʹ fragment and PstI/BamHI for the 3ʹ fragment. The pLPBLP–*knkA* KO vector was linearized with ScaI and transformed into Ax2 cells.

*bcas3* knockout vector: 5ʹ and 3ʹ fragments of the *bcas3* genomic region (Fig. S1B) were amplified with primer pairs *bcas3-f1/bcas3-r1* and *bcas3-f2/bcas3-r2*, respectively, and sub-cloned into pJet1.2/blunt. The 5ʹ fragment was inserted into pLPBLP using PstI/BamHI, followed by the 3ʹ fragment, which was excised with HindIII/XhoI and inserted into the HindIII/SalI digested vector. The BamHI/KpnI knockout fragment was excised and transformed into Ax2 cells.

[*act15*]-*knkA-YFP*: the *knkA* coding region was amplified using primers *knkA-f4/knkA-r4*, and after sub-cloning in pJet1.2/blunt, digested with inserted HindIII/EcoRI and ligated into HindIII/EcoRI digested pDV-CYFP [48]. The resulting [*act15*]*-knkA-YFP* fragment was excised using SalI/XhoI and ligated into SalI/XhoI digested pExp4-Hyg [12].

*knkA-YFP* knock-in: *knkA-YFP* was amplified from [*act15*]*-knkA-YFP* using primers *knkA-f4* and *YFP-r1* and sub-cloned into pJet1.2/blunt. A *knkA-YFP* fragment starting at nt 284 of *knkA* was excised using BamHI/NdeI and ligated into BamHI/NdeI digested pLPBLP. In vector pLPBLP, the blasticidin selection cassette is flanked by *LoxP* sequences, allowing it to be excised by transformation with Cre-recombinase in order to re-use blasticidin as a selection marker [47]. This plasmid was then digested using HindIII/SalI, and a fragment of the *knkA* 3ʹ UTR, which was amplified from Ax2 gDNA using primer pair *knkA-f5/knkA-r3* and digested with HindIII/XhoI, was inserted. A BamHI/KpnI fragment was excised and transformed into Ax2 and *bcas3^−^*.

*knkA-mCherry*-knock-in: The YFP-Bsr fragment was excised from the *knkA-YFP* knock-in vector using EcoRI/NdeI and replaced by a mCherry fragment that was amplified from pDM1208 [49] with primer pair *mCherry-f/mCherry-r* and digested with EcoRI/NcoI. The resulting vector was cut with NcoI and, after filling in with Klenow fragment, the Bsr cassette, which was excised from pLPBLP using SmaI, was inserted by blunt-end ligation. The KpnI/BamHI fragment of the resulting *knkA-mCherry*-KI plasmid was used for the transformation of Ax2.

*bcas3-YFP* knock-in: *bcas3* gDNA was amplified using *bcas3-f2* and *bcas3-r2* and cloned into pJet1.2/blunt. The XbaI site of pJet1.2 was deleted by partially digesting the plasmid, filling in with Klenow fragment and religation to yield *bcas3*-pJet1.2/blunt. The Bsr cassette was excised from pLPBLP using SalI/PvuII and cloned into SpeI/EcoRV digested vector pDd-CYFP [48], and the resulting YFP-Bsr fragment was excised using SpeI and introduced into an XbaI site that is internal to *bcas3* in *bcas3*-pJet1.2/blunt. The resulting plasmid was linearized using NotI and transformed into Ax2 and into *knkA*-mCherry knock-in cells, from which the Bsr cassette was removed by transformation with pA15NLS.Cre [47].

[*act15*]*-myc-bcas3*: the *bcas3* coding sequence was amplified from Ax2 gDNA using *bcas3-f3/bcas3-r2*, sub-cloned into pJet1.2/blunt, and after HindIII/XhoI digesting ligated into HindIII/XhoI digested pDV-NYFP [48].

[*bcas3p*]-RFP-*bcas3* and *bcas3*^LAAG^: pDV-mRFPmars was created by amplifying mRFP mars from pmRFPmars [48] using primer pair RFP-f/RFP-r. After subcloning, the HindIII/SpeI digested PCR product was ligated into HindIII/SpeI digested pDV-NYFP, replacing YFP. The 993 bp 5’UTR  of *bcas3* that covers most of the 5’ intergenic region was amplified from Ax2 gDNA using primer pair *bcas3prom-f/bcas3prom-r*, digested with SalI/BamHI, and ligated into SalI/BamHI digested pDV-mRFPmars, upstream of mRFPmars. The *bcas3* coding region was excised with NheI/XhoI from [*act15*]-*myc-bcas3* (see above) and ligated  into XbaI/XhoI digested bcas3p-mRFPmars. The resulting [*bcas3p*]-mRFP-*bcas3* fragment was transferred to pExp4-Hyg using SalI/XhoI. K541A and R542A mutations in *bcas3* were introduced by amplifying 5' and 3' *bcas3* fragments using *bcas3-f3/bcas3mut-r* and *bcas3mut-f/bcas3-r4* primer pairs, respectively, and annealing the amplicons. The *bcas3* segment that harboured the mutation was then amplified using primers *bcas3-f3/bcas3-r4* and the EcoRI digested product was used to replace the EcoRI flanked segment of [*bcas3p*]-RFP-*bcas3*.

YFP-SKL: DNA oligonucleotides SKL-up and SKL-down were annealed and inserted into XbaI/XhoI digested pDV-NYFP. The YFP-SKL fusion was excised with BglII/XhoI and inserted into BglII/XhoI digested pDV-NYFP-Hyg [11].

[*act15*]-*RFP-atg8-Hyg*: The [*act15*]-*RFP-atg8* fragment was excised from pA15/RFP-Apg8 [35] using EcoRV/BglII, filled in with Klenow fragment and ligated into pExp4-Hyg [12] that was cut by SalI/XhoI and filled in.

pLPBLP, CnxA-GFP, Gol-GFP and GppA-GFP were obtained from the Dicty Stock Center http://dictybase.org/StockCenter/StockCenter.html.

*His-Dl-bcas3* and *His-Dl-bcas3*^LAAG^: To create *His-Dl-bcas3*, nt 7–1866 was amplified from *Dictyostelium lacteum* cDNA using primers *Dl-bcas3-f1* and *Dl-bcas3-r1*, and cloned into pET28a (Merck, 69864) using BamHI and XhoI. To create the LAAG mutation, 5ʹ and 3ʹ fragments of *Dl-bcas3* were amplified using *Dl-bcas3-f2/Dl_bcas3_mut-r* and *Dl_bcas3_mut-f/Dl-bcas3-r2* respectively, annealed and amplified using primer pair *Dl-bcas3-f2/Dl-bcas3-r2*. The KpnI/ScaI fragment that contained the mutation was cloned into KpnI/ScaI digested His-Dl-bcas3 to create *His-DI-bcas3*^LAAG^.

### Immunoprecipitation and mass spectrometry

To identify KnkA-interacting proteins, dissociated *knkA^−^* and *knkA^−^*/A15-KnkA-YFP slug cells were suspended at 5 × 10^7^ cells/ml in IPH buffer (20 mM HEPES, pH 7.5, 150 mM NaCl, 1% NP40 alternative (Calbiochem, 492016), 0.1% Triton X-100 (Fluka, BP151-100), containing 1 mM di(N-succinimidyl)3,3ʹ-dithiodipropionate (DSP; Thermo Scientific, 22586), EDTA-free cOmplete™ protease inhibitors (Roche, 11873580001), 2 mM benzamidine (Sigma-Aldrich, 434760) and 0.2 mM TLCK (Sigma-Aldrich, T7254), and incubated on ice for 2 h. After quenching DSP by 30 min incubation with 50 mM Tris, pH 8 (final concentration), the lysate was centrifuged at 16000 x g for 10 min. The supernatant was incubated with 20 µl GFP-Trap-agarose beads (Chromotek GmbH gta-10) at 4ºC overnight, washed with IPT buffer (50 mM Tris, pH 8, 150 mM NaCl, 1% NP40 alternative, 0.1% Triton X-100, 2 mM EDTA), and eluted with SDS buffer without thiol reagents. Proteins were size-fractionated on SDS-PAGE and the fraction larger than monomeric KnkA-YFP were excised from the gel and analyzed by LC-MS-MS mass spectrometry. Proteins were identified and quantified using MaxQuant [50] https://www.maxquant.org/. Statistical analysis of proteins enriched in *knkA^−^*/A15-KnkA-YFP over *knkA^−^* immunoprecipitates was performed using Perseus [51] https://www.maxquant.org/. Where LFQ (label-free quantification) intensity was 0, an arbitrarily low value of 2 was used to calculate the log2 ratio of protein abundance. The FDR was calculated with the S2 constant set at 0.2.

Immuno-precipitation of KnkA-YFP and MYC-Bcas3 was performed as outlined above, except that cells were lysed in IPT buffer without DSP and proteins were pulled down using anti-GFP antibodies (Roche, 11814460001) or anti-MYC 9E10 antibodies (Invitrogen, 132500), and Dynabeads Protein G (Invitrogen, 10003D). Proteins were detected by western blotting [11] using the same anti-GFP or anti-MYC 9E10 antibodies.

### Confocal microscopy

To analyze cellular localization of KnkA and Bcas3 isoforms with fluorescent tags, cells were cultured in Loflo medium (Formedium, LF0501) for 6 h and then starved on non-nutrient agar to induce aggregation or on water agar under unidirectional light to induce slug formation. Slug pre-stalk cells were prepared by collecting the anterior 25% of slugs into 20 mM K-phosphate (16 mM KH_2_PO_4_ (Merck, P5655) and 4 mM K_2_HPO_4_ (Merck, 1551128), pH 6.2) (KK2), which were dissociated briefly by vigorous pipetting, as were cell aggregates. Cells were deposited in a glass-bottomed dish, overlayed with ~0.75 mm of 1% agarose and imaged using a Leica SP8 confocal microscope. For analysis of RFP-GFP-Atg8, z series of images in 0.3–0.5 µm steps were captured and a maximum intensity projection of these images was used to quantify fluorescent vesicles.

### Expression and PIP binding of Dl-Bcas3 and Dl-Bcas3^LAAG^

Both the *His-Dl-bcas3* and *His-Dl-bcas3*^LAAG^ plasmids were transformed into *E. coli* BL21(DE3)pLysS (BioDynamics Laboratory Inc, DS260) and expression was induced with 0.1 mM IPTG (Takara Bio, 9030). Total protein was isolated by sonicating the bacteria in binding buffer (300 mM NaCl, 50 mM sodium phosphate, pH 7.0) containing EDTA-free cOmplete™ protease inhibitors. The his-tagged Bcas3 proteins were purified by incubation with Talon Metal Affinity resin (Clontech, 635501), washing of the resin with 10 mM imidazole (Merck, 137098) in binding buffer and elution with 200 mM imidazole in binding buffer with protease inhibitors. The eluate was concentrated using Amicon Ultracell 30K (Merck Millipore, UFC903008) and dialyzed with TBS (50 mM Tris-Cl, 150 mM NaCl, pH 7.5). PIP strips (Echelon Biosciences, P-6001) were incubated with approximately 200 ng of His-Dl-Bcas3^LKRG^ or His-Dl-Bcas3^LAAG^ in TBS containing 0.05% Tween 20 (VWR, 437082Q) and 3% BSA (Sigma-Aldrich, A7906), and after washing with HRP-conjugated anti-His antibody (Sigma-Aldrich, A7058), followed by HRP detection using ECL Select Western Blotting Detection Reagent (GE Healthcare, RPN223).

### Statistical analysis

Descriptive statistics (mean, standard deviation and standard error) were calculated in Sigmaplot v.14 (Systat Software Inc, sp14acad). Significant differences between mean experimental values obtained after different treatments or in different cell lines were determined by a t-test in Sigmaplot, when data were normally distributed and by a Mann-Whitney Rank Sum test, when they were not, using a two-tailed P-value of 0.05 as the threshold. Statistical analysis of significant protein-enrichment after immunoprecipitation and mass-spectrometry was performed using Perseus [51]. The FDR (false discovery rate) was calculated with the S2 constant set at 0.2. Significant differences in total, red, and yellow puncta between RFG-GFP-Atg8 transformed wild type, *knkA-* and *bcas3-* cell lines were determined with Wilcoxon’s rank-sum test in R [52].

## Supplementary Material

Supplemental Material

Supplemental Material

## Data Availability

The mass spectrometry data generated in this work are listed in supplemental data file Data1_KnkA_coIP_MaxQuant.xlsx. The plasmid constructs and mutants that were generated in the course of this work are deposited in the *Dictyostelium* Stock Center http://dictybase.org/StockCenter/StockCenter.html.
