# Supplementary material for: The proppin Bcas3 and its interactor KinkyA localize to the early phagophore and regulate autophagy
Source: Autophagy. 2020 Mar 1;17(3):640–55. doi: 10.1080/15548627.2020.1725403 (PMC8032249; doi:10.1080/15548627.2020.1725403)
Supplement: Supplemental Material [file KAUP_A_1725403_SM0609.docx]

**SUPPLEMENT**

**Contents**

[Figure S1. Knockout and knockin constructs and diagnosis 3](#_Toc28960961)

[Figure S2. Alignment and annotated phylogenetic tree of KnkA homologs 5](#_Toc28960962)

[Figure S3. Stalk and spore gene expression 6](#_Toc28960963)

[Figure S4. Immuno-precipitation of Bcas3 with KnkA-YFP 7](#_Toc28960964)

[Figure S5. Proppin alignment and phylogenetic distribution of Bcas3 homologs 8](#_Toc28960965)

[Figure S6. Colocalization of KnkA with Bcas3 wild-type and mutant protein and with organelle markers 10](#_Toc28960966)

[Figure S7. RFP-GFP-Atg8 expression in aggregative wild-type, *knkA^-^* and *bcas3-* cells 11](#_Toc28960967)

[Figure S8. RFP-GFP-Atg8 expression in wild-type, *knkA^-^* and *bcas3^-^* pre-stalk and pre-spore cells 12](#_Toc28960968)

[Table S1. Oligonucleotides used in this work 13](#_Toc28960969)

[Table S2. Top-enriched proteins that were co-immuno-precipitated with KnkA-YFP 14](#_Toc28960970)

[Table S3. RFP-GFP-Atg8 quantification summary. 14](#_Toc28960971)

[Supplemental references 14](#_Toc28960972)

**
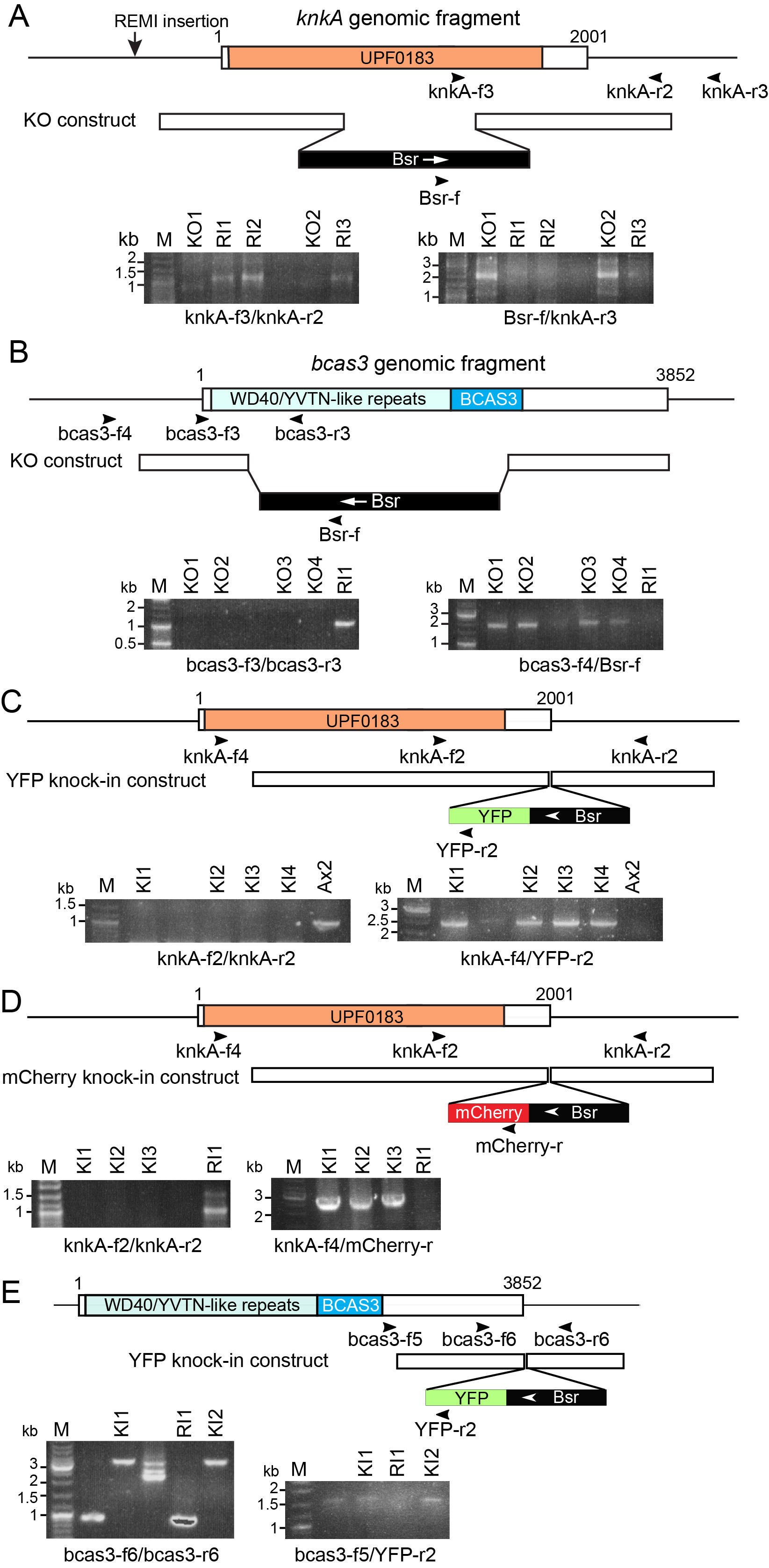
**

# Figure S1. Knockout and knockin constructs and diagnosis. Panels show genomic fragments that harbor the targeted genes and a schematic of the knockout or knockin construct with the positions of the primers (arrowheads) that were used to diagnose a homologous recombination event in transformed clones. Gel images show the PCR products amplified from genomic DNAs isolated from some knockout (KO), knockin (KI) or random integrant (RI) clones for each construct or Ax2 cells when no RI clones were obtained. (A) *knkA* knockout. Primer pair knkA-f3/knkA-r2 (Table S1) amplifies a 1.1-kb fragment from the RI but not KO gDNAs, whereas primer pair Bsrf/knkA-r3 amplifies a 1.9-kb fragment from KO gDNAs only. (B) *bcas3* knockout. Primer pair bcas3-f3/bcas3-r3 amplifies a 0.9-kb fragment from RI gDNAs, while bcas3-f4/Bsr-f amplifies a 1.8-kb fragment from KO gDNAs. (C) *knkA-YFP* knockin. Primer pair knkA-f2/knkA-r2 amplifies a 1-kb fragment from Ax2 gDNA, while knkA-f4/YFP-r2 amplifies a 2.1-kb fragment from KI gDNAs only. (D) *knkA-mCherry* knockin. Primer pair knkA-f2/knkA-r2 amplifies 1 kb from RI gDNAs, and knkA-f4/mCherry-r amplifies 2.7-kb fragment from KI gDNAs. (E) *bcas3-YFP* knockin. Primer pair bcas3-f6/bcas3-r6 amplifies 0.9-kb and 3-kb fragments from RI and KI gDNAs, respectively. Primer pair bcas3-f5/YFP-r2 amplifies a 1.6-kb fragment from KI gDNAs.


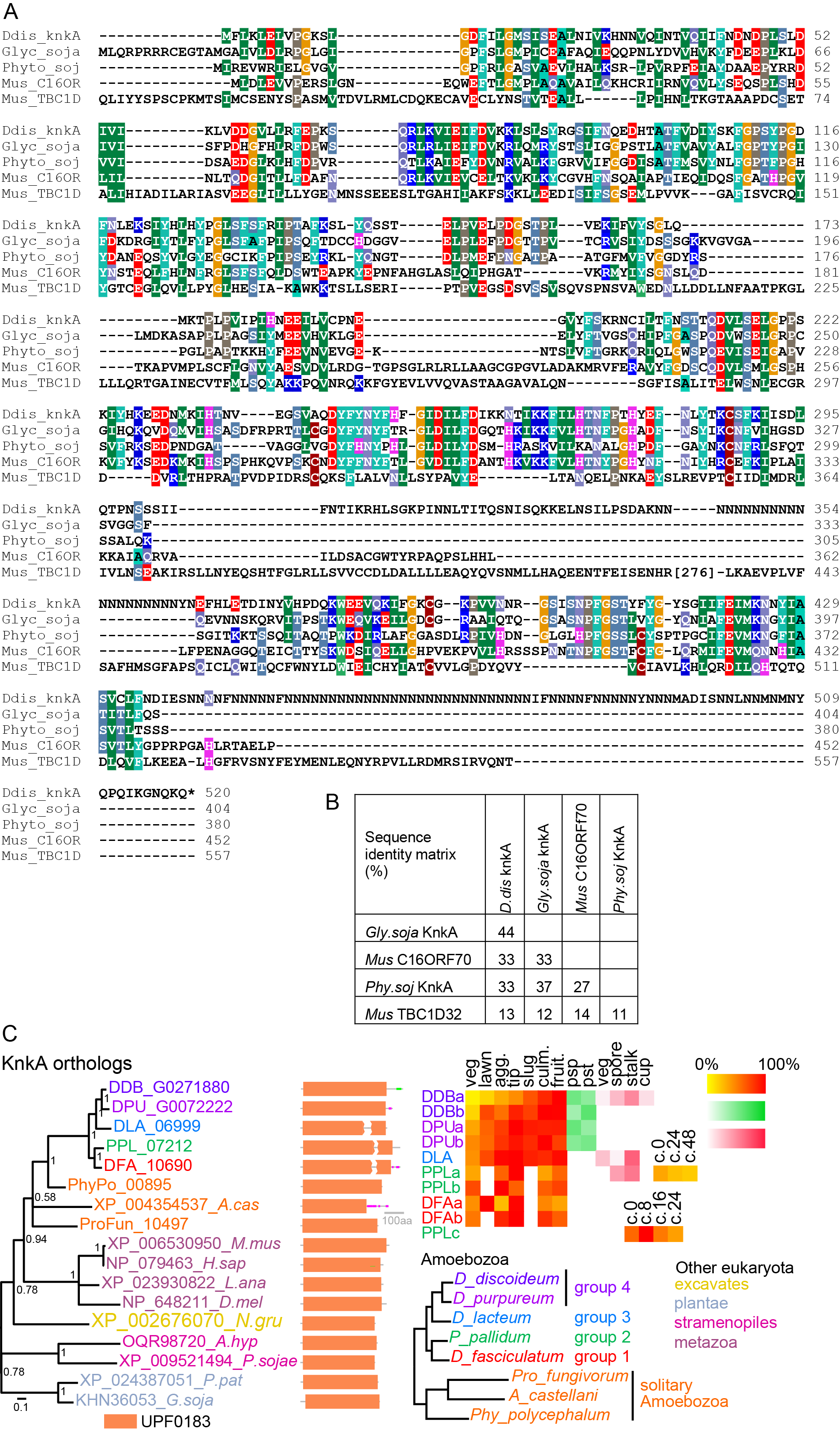


# Figure S2. Alignment and annotated phylogenetic tree of KnkA homologs (A) Protein sequences of *D. dis* KnkA, mouse C16ORF70, their closest homologs in the plant *Glycine soja* and the stramenopile *Phytophtera sojae* were aligned with the related mouse protein mouse TBC1D32 (broad-minded). Conserved residues across three or more proteins are shaded in color. (B) Pairwise sequence identities between the aligned proteins. (C) KnkA homologs were identified by separate BlastP searches of major eukaryote divisions in Genbank and from taxon group-representative *Dictyostelium* proteomes. Protein sequences for a number of top hits from each division were aligned using ClustalW [1] and a phylogenetic tree was inferred by Bayesian inference [2]. The tree is annotated with the domain architecture of the proteins as determined by SMART [3] and for the dictyostelid genes with heat maps of the developmental expression profiles (a,b replicate experiments), relative expression in pre-stalk and prespore cells of slugs, and relative expression in growing cells and the mature stalk, spore and cup cells of fruiting bodies [4,5]. For *P. pallidum* regulation during development to cysts is also shown (time points in hours, preceded by c).


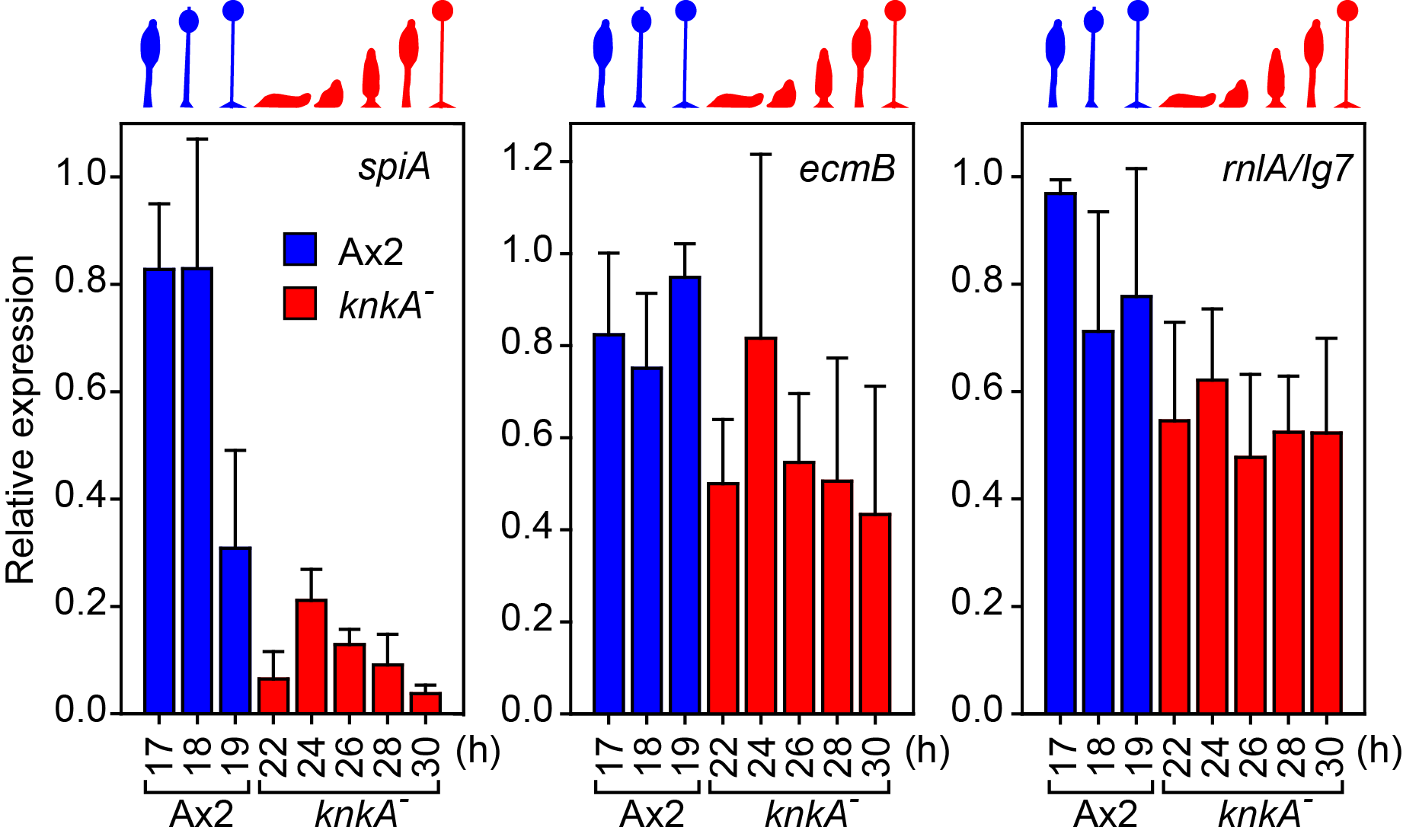


# Figure S3. Stalk and spore gene expression. *knkA^-^* cells are significantly delayed in post-aggregative development. To compare stalk and spore gene expression during fruiting body formation, *knkA^-^* structures were therefore harvested at time points at which their development was morphologically equivalent to that of Ax2 cells. RNAs were isolated and RT-qPCR was performed with primers complementary to the spore gene *spiA*, the stalk gene *ecmB* and the constitutively expressed gene *rnlA*/*ig7*. Means and SD of three experiments with technical duplicates are presented.


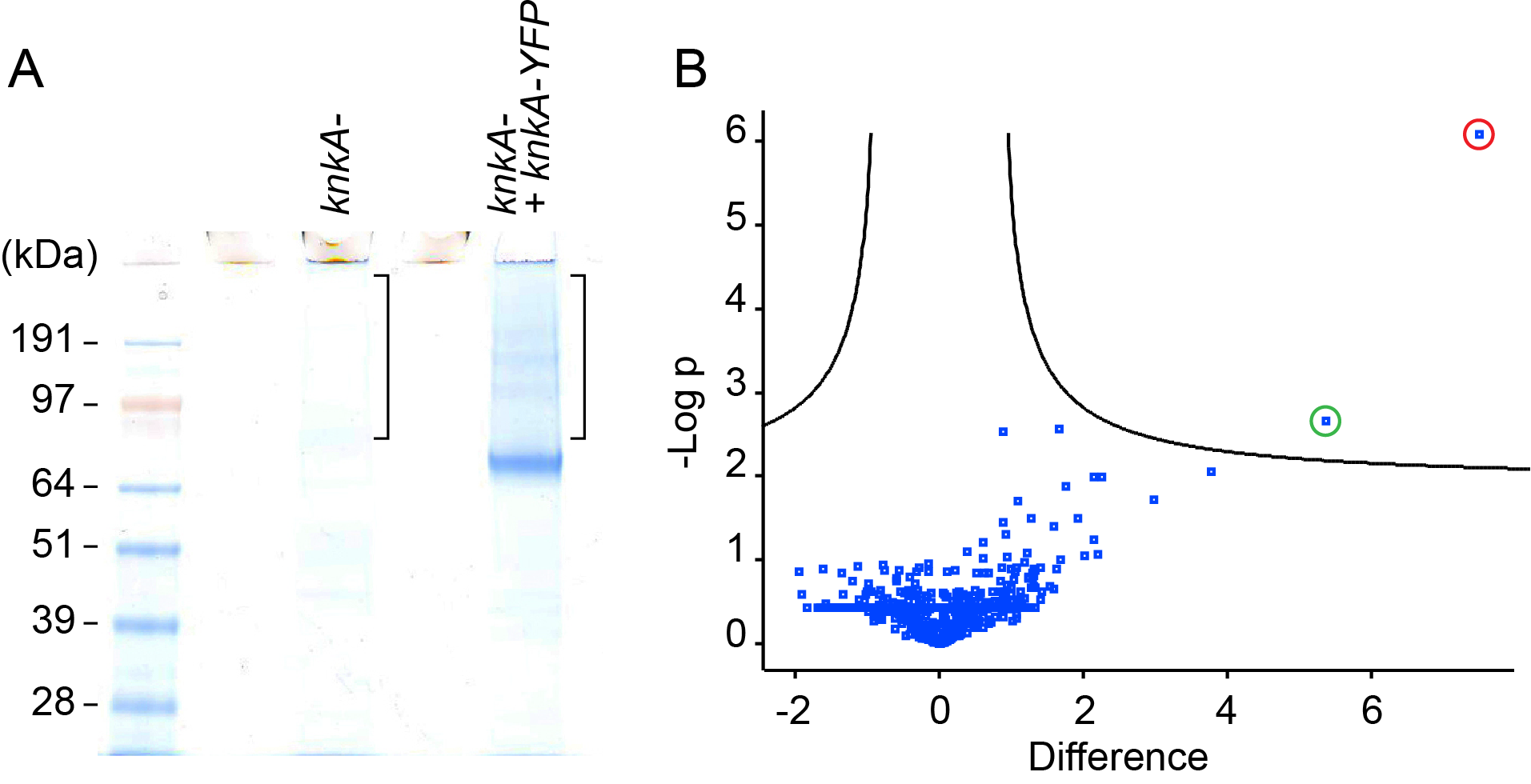


# Figure S4. Immunoprecipitation of Bcas3 with KnkA-YFP. (A) Developed structures (early slug to Mexican hat stage) of *knkA^-^*/[*act15*]-KnkA-YFP (Hyg) and *knkA^-^* were lysed and proteins were cross-linked with 1 mM DSP. Lysate without cross-linking was prepared as a control. Lysates were immuno-precipitated using GFP-trap agarose and bound material was extracted with SDS sample buffer without beta-mercaptoethanol (which cleaves the cross-linker). The samples were size-fractionated and the top regions of the lanes, which contain the cross-linked proteins, were subjected to LC-MS-MS mass spectrometry. Protein identification and quantification were performed using MaxQuant (see Table S2 and Supplemental spreadsheet Data1_knkA_coIP_MaxQuant.xlsx). (B) Statistical analysis of the fold-enrichment of proteins pulled down with KnkA-YFP in three experiments. The solid line shows statistical support at False Discovery Rate (FDR)=0.05. Bcas3 and KnkA are circled in green and red, respectively.


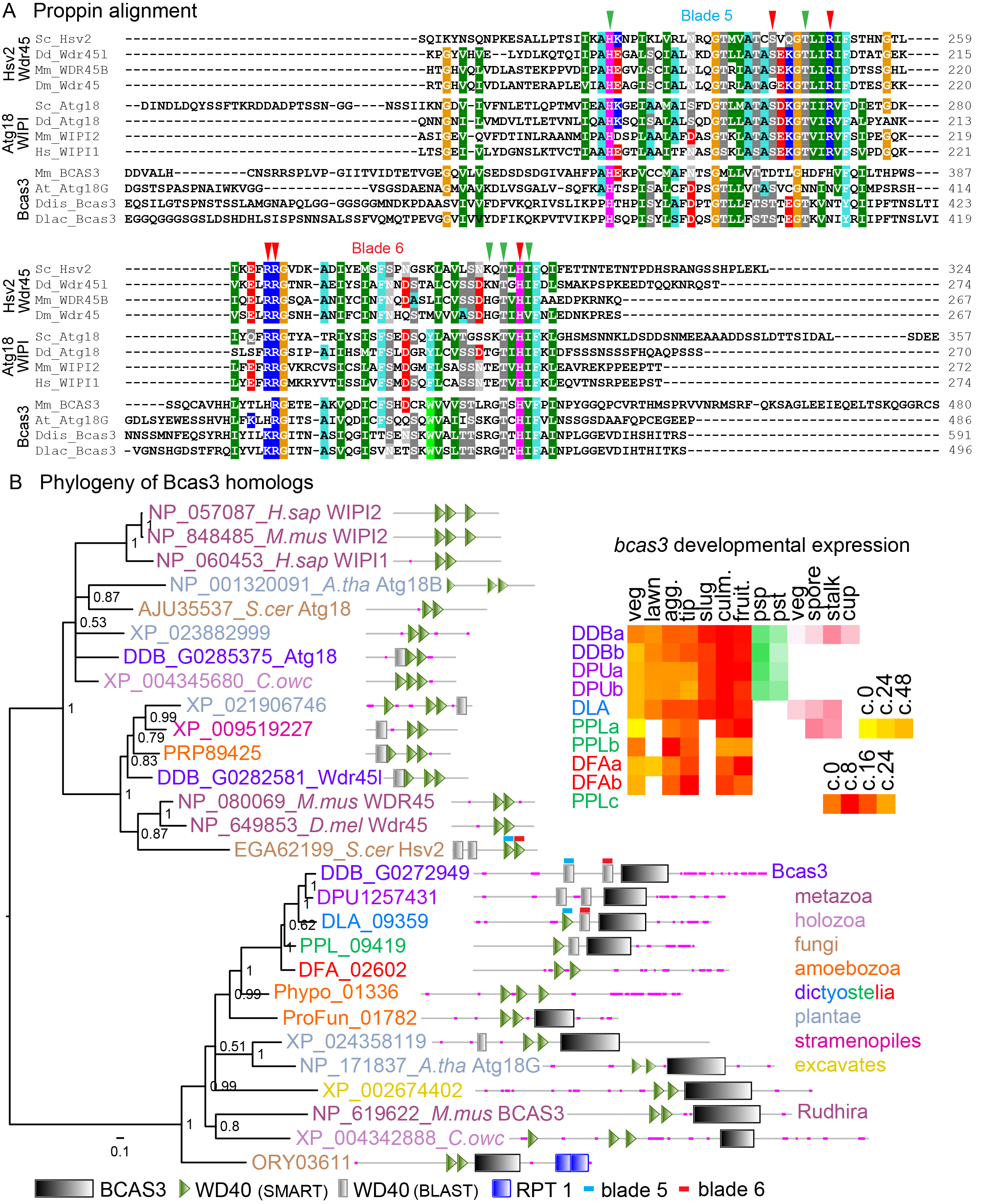


**Figure S5.** Proppin alignment and phylogenetic distribution of Bcas3 homologs. (**A**) Alignment. A selection of Wdr45 and Atg18 homologs from model organisms were aligned with the structurally well-characterized proppin Hsv2 from yeast and a selection of Bcas3 proteins. Only blades 5 and 6, which contain the proppin inositol phospholipid-binding sites, are shown. Amino-acids with essential and supporting roles in phospholipid binding are indicated by red and green arrows, respectively [6,7]. (**B**) Phylogeny*.* Genomes representative of the major eukaryotic divisions were queried by BLASTp for the presence of homologs to *D. discoideum* Bcas3. Proteins were aligned and a phylogenetic tree was constructed by Bayesian inference. The tree was annotated with the functional domain architecture of the proteins as detected by SMART and for *Dictyostelium* Bcas3 orthologs with the developmental expression profile and cell-type specificity of the transcripts. Note that not all WD40 repeats in the proteins are detected by the SMART and BLAST models for this domain.


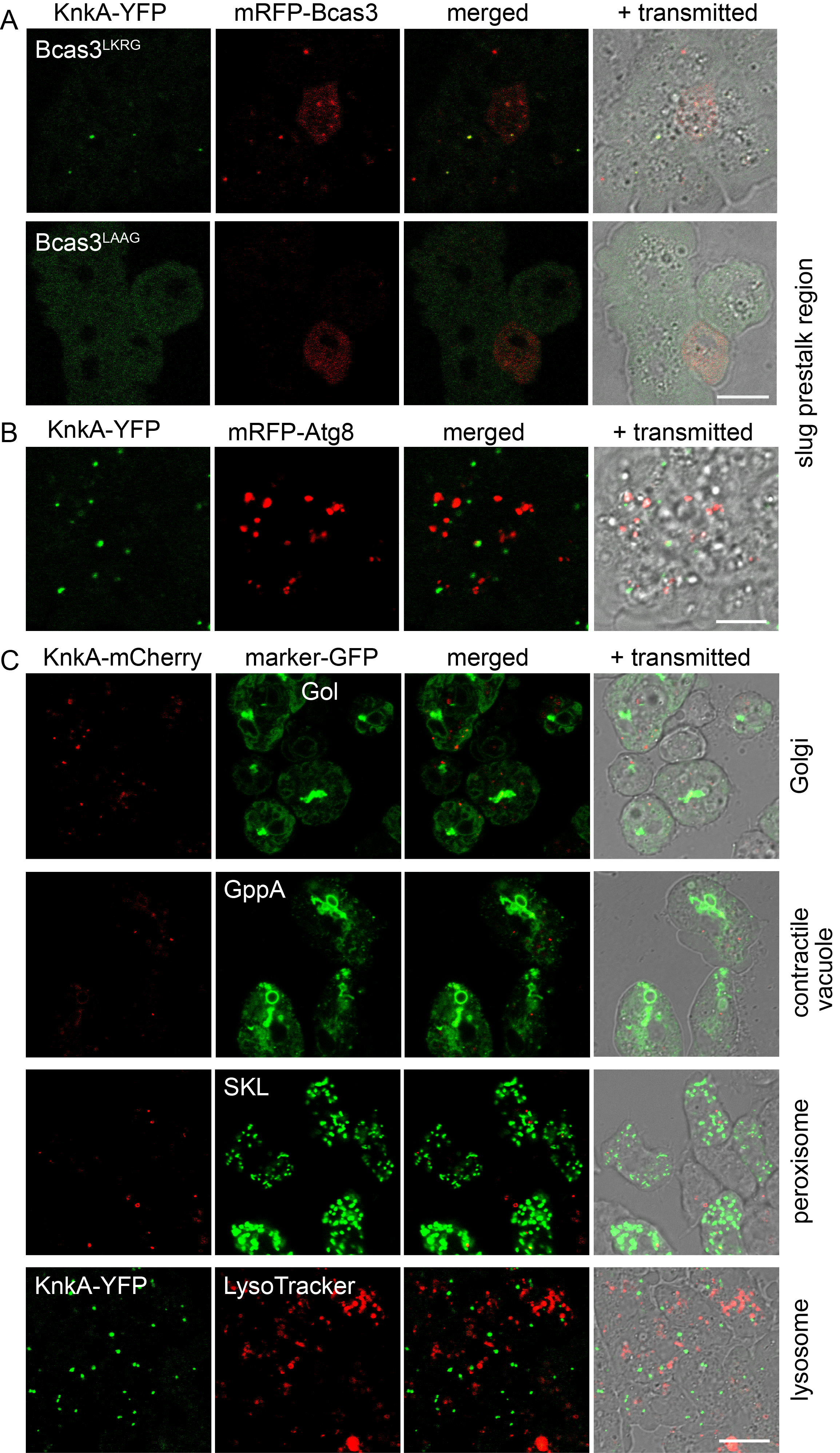


# Figure S6. Colocalization of KnkA with Bcas3 wild-type and mutant protein and with organelle markers. (A) KnkA recruitment in pre-stalk cells. Dissociated pre-stalk regions of *bcas3^-^*/KnkA-YFP knockin cells, transformed with [*bcas3*]*-mRFP-bcas3*^LKRG^ (wild-type) or [*bcas3*]*-mRFP-bcas3*^LAAG^, were imaged by confocal microscopy. Individual and merged images are shown, the latter also merged with the transmitted light image. (B) KnkA and Atg8 in pre-stalk cells. Dissociated pre-stalk regions of Ax2 *knkA-YFP* knockin cells transformed with mRFP-Atg8 were imaged as in panel A. (C) KnkA and organelle markers. Dissociated aggregates of Ax2 *knkA-mCherry* knockin cells, transformed with the indicated GFP-tagged markers for Golgi, contractile vacuoles or peroxisomes or KnkA-YFP knockin cells stained with LysoTracker Red were imaged as in panel (A) Bars: 10 µm.


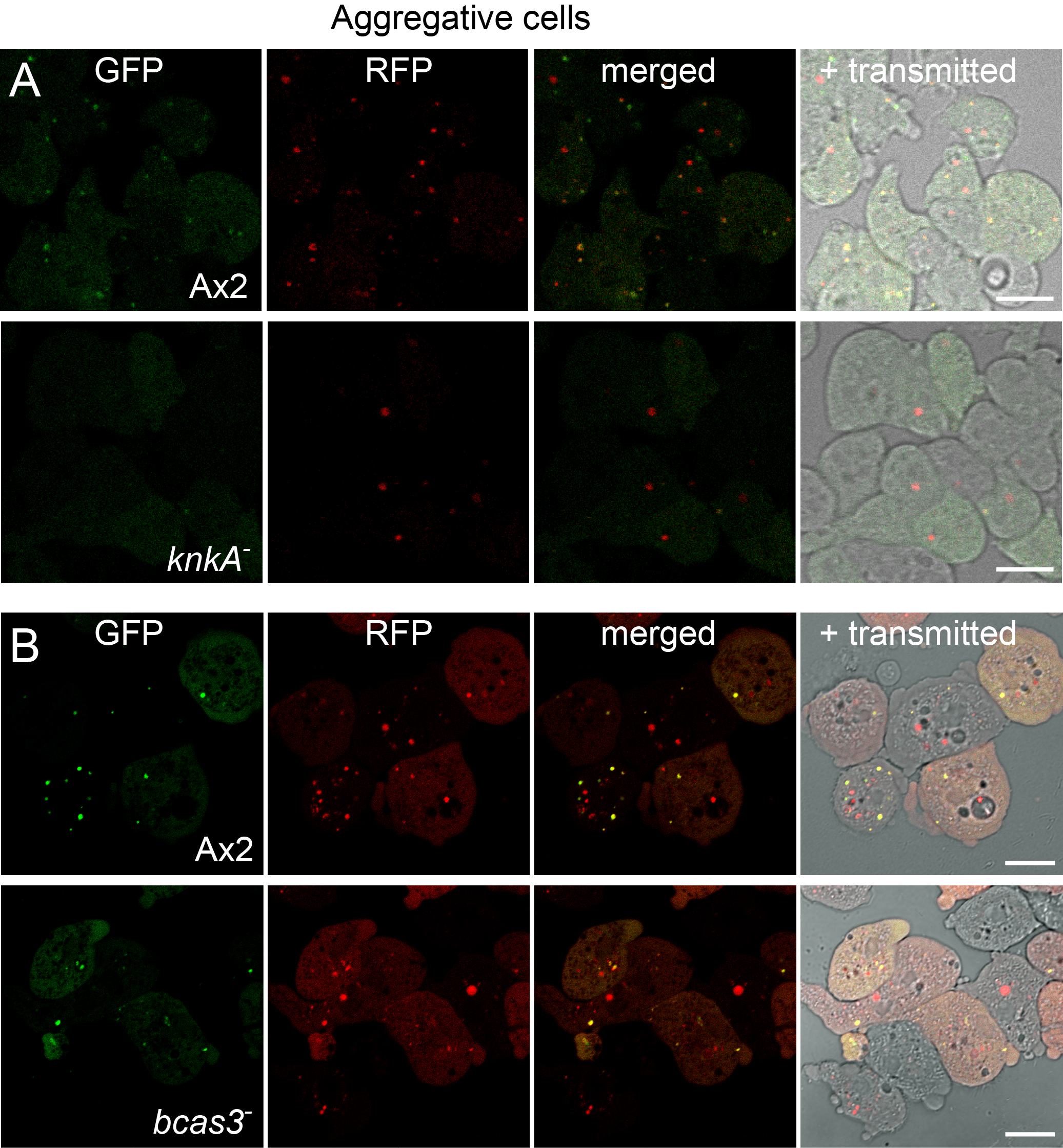


# Figure S7. RFP-GFP-Atg8 expression in aggregative wild-type, *knkA^-^* and *bcas3-* cells. Dissociated aggregates of Ax2 and *knkA^-^* cells (A) or Ax2 and *bcas3^-^* cells (B), transformed with RFP-GFP-Atg8, were imaged by confocal microscopy for RFP and GFP fluorescence. Bars: 10 µm.


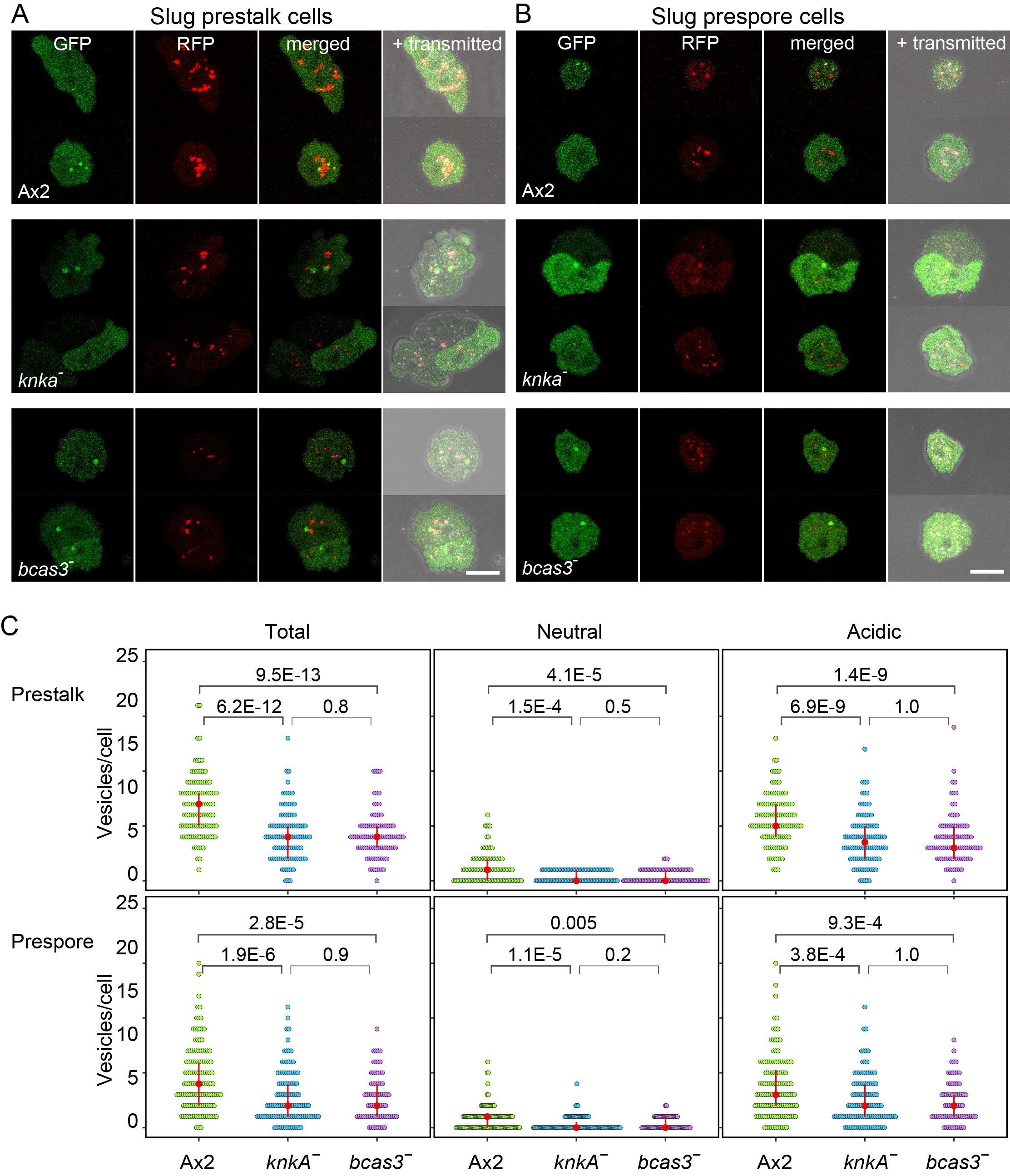


# Figure S8. RFP-GFP-Atg8 expression in wild-type, *knkA^-^* and *bcas3^-^* pre-stalk and prespore cells. (A) Ax2, *knkA^-^* and *bcas3^-^* cells transformed with RFP-GFP-Atg8 were developed into migrating slugs. The anterior 25% (pre-stalk) or posterior 75% (pre-spore) regions of slugs were isolated with a needle, dissociated and imaged by confocal microscopy for RFP and GFP fluorescence. Bars: 10 µm. (B) Quantification. Numbers of vesicles in each cell that showed both RFP and GFP fluorescence (neutral), or only RFP fluorescence (acidic) were counted, and the sum of neutral and acidic vesicles (total) was calculated. Red circles and red bars represent the median and quartile of each dataset, respectively. The numbers above the brackets are P-values of significant differences between Ax2 and *knkA^-^* cells (Wilcoxon rank-sum test). See Table S3 for means and SE values and numbers of cells analyzed per cell type.

# Table S1. Oligonucleotides used in this work.

| **Name** | **Sequence** |
| --- | --- |
| knkA-f1 | GTCGACCAACATAAAACAACCTATTCTG |
| knkA-f2 | CTGCAGGGAAAGTGCGGAAAACCAG |
| knkA-f3 | CTGATCAAAAATGGGAAGAGG |
| knkA-f4 | AAGCTTTTTTTAAAATTAGAACTTGTACCAGG |
| knkA-f5 | AAGCTTCTCCTTTGCTATTCCTTTAATG |
| knkA-r1 | AAGCTTACTGGTAGCTCTGTGCTAC |
| knkA-r2 | GGATCCTCAACTGCTTCTGTTGATCC |
| knkA-r3 | GTTCCATTAGCAGGTACAGG |
| knkA-r4 | GAATTCTTGTTTTTGGTTTCCTTTAATTTGTG |
| bcas3-f1 | GGATCCACCCAATCATTGATTGGATTG |
| bcas3-f2 | AAGCTTTCTATGGAGGGTGGTAGTG |
| bcas3-f3 | AAGCTTGAACAAAAATTAATTTCAGAAGAGGATCTCAACGCTAGCTACCAAAATGCATTAAATCATAC |
| bcas3-f4 | CTACAAAACTTTAAAAGAGGTGGC |
| bcas3-f5 | TGCAATCTGGGGTGTACC |
| bcas3-f6 | CCAGCTTATCCACATTTAGG |
| bcas3-r1 | CTGCAGCCATCTCTACATGATAACAATTCC |
| bcas3-r2 | CTCGAGTTATTTTCTAGATTTCTTTGAGGAACC |
| bcas3-r3 | TCCAACGAGATCCCAATGC |
| bcas3-r4 | CCGTTACCGTTATTTATAGTTATAGG |
| bcas3-r6 | GAAGCCAACTGAAAGGGGTC |
| bcas3prom-f | GTCGACTGGGTTTACCCAAATTAATTTC |
| bcas3prom-r | GGATCCCATTGTTTTTATTATACTTTTGTTG |
| bcas3mut-f | GACATATTTATATTTTAGCAGCTGGTATTACAAATGC |
| bcas3mut-r | GCATTTGTAATACCAGCTGCTAAAATATAAATATGTC |
| Bsr-f | GTGGTAAGTCCTTGTGG |
| YFP-r1 | GGGCATATGTTACAGCTCGTCCATGCCGAG |
| YFP-r2 | GAACTTCAGGGTCAGCTTGCCG |
| mCherry-f | GAATTCATGGTTTCAAAAGGTGAAG |
| mCherry-r | CCATGGTTATTTATATAATTCATCCATACCAC |
| SKL_up | CTAGAGAGCTGTCAAAATTATAAC |
| SKL_down | TCGAGTTATAATTTTGACAGCTCT |
| RFP-f | AAGCTTATGGCATCATCAGAAGATG |
| RFP-r | ACTAGTTGCACCTGTTGAATGTC |
| Dl_bcas3_f1 | GCGGATCCAACCTAAATGTAAATAGTAATAATATAACAGG |
| Dl_bcas3_r1 | CCCTCGAGTTATGTAAATGGTGTATTCATTGCC |
| Dl_bcas3_f2 | CAAGGAGGAGGTTCAGGG |
| Dl_bcas3_r2 | GGTCTTGATTGGGAGGTGG |
| Dl_bcas3_mut-f | GACAAATTTATGTACTCGCAGCTGGTATTACCAATGC |
| Dl_bcas3_mut-r | GCATTGGTAATACCAGCTGCGAGTACATAAATTTGTC |

**Table S2.** Top-enriched proteins that were co-immunoprecipitated with KnkA-YFP**.**

| Gene names | Description | Enrichment log_ratio | | | knockout phenotype |
| --- | --- | --- | --- | --- | --- |
|  |  | Exp.1 | Exp.2 | Exp.3 |  |
| DDB_G0271880 | KnkA | 27.4 | 27.8 | 27.3 | defective sporulation |
| DDB_G0272949 | Bcas3 | 24.2 | 26.8 | 25.1 | defective sporulation |
| *ndrA* | NDR kinase | 22.4 | 23.4 | 20.6 | decreased engulfment |
| DDB_G0285363 | contains RNA recognition motif | 21.9 | 21.3 | 20.6 | N/A |
| DDB_G0278499 | protein phosphatase 2C | 20.9 | 22.9 | 19.5 | N/A |
| *phlp1* | phosducin-like protein | 21.2 | 21.2 | 20.3 | no aggregation |
| DDB_G0285341 | unknown | 21.9 | 20.5 | 20.2 | N/A |
| DDB_G0287281 | citrate synthase | 19.7 | 21.7 | 20.1 | N/A |
| DDB_G0275365 | heterogeneous nuclear ribonucleoprotein L | 20.7 | 20.7 | 19.4 | N/A |
| DDB_G0268884 | ortholog of (E) *coli* OsmC | 20.0 | 20.9 | 19.7 | N/A |
| DDB_G0275227 | FKBP-type peptidylprolyl cis-trans isomerase | 20.5 | 20.2 | 19.7 | N/A |
| DDB_G0286603 | glucosamine-fructose-6-phosphate aminotransferase | 19.2 | 21.7 | 18.9 | N/A |
| *cbpD1* | calcium-binding protein | 19.7 | 19.6 | 20.3 | N/A |

Proteins detected by mass spectrometry that was enriched in cross-linked *knkA^-^*/KnkA-YFP over *knkA^-^* lysates, immuno-precipitated with GFP-trap-agarose. The full mass spectrometry dataset is listed in Supplemental Data1_knkA_coIP_MaxQuant.xlsx.

**Table S3**. RFP-GFP-Atg8 quantification summary.

| Cell type | Vesicles | Ax2 | (nc/ne/nt) | *knkA^-^* | (nc/ne/nt) | *bcas3^-^* | (nc/ne/nt) |
| --- | --- | --- | --- | --- | --- | --- | --- |
| Aggregating | Total | 6.3 ± 0.3 | (149/4/2) | 3.4 ± 0.2 | (118/3/2) | 2.8 ± 0.2 | (157/2/2) |
|  | Neutral | 4.8 ± 0.3 |  | 1.9 ± 0.2 |  | 1.5 ± 0.1 |  |
|  | Acidic | 1.5 ± 0.1 |  | 1.5 ± 0.1 |  | 1.3 ± 0.1 |  |
| Slug  Pre-stalk | Total | 6.8 ± 0.3 | (112/6/2) | 4.2 ± 0.2 | (100/3/1) | 4.2 ± 0.2 | (93/3/2) |
|  | Neutral | 1.1 ± 0.1 |  | 0.5 ± 0.1 |  | 0.4 ± 0.1 |  |
|  | Acidic | 5.6 ± 0.2 |  | 3.8 ± 0.2 |  | 3.8 ± 0.2 |  |
| Slug  Pre-spore | Total | 4.5 ± 0.3 | (124/6/2) | 2.8 ± 0.2 | (107/3/1) | 2.7 ± 0.3 | (69/3/2) |
|  | Neutral | 0.8 ± 0.1 |  | 0.3 ± 0.1 |  | 0.4 ± 0.1 |  |
|  | Acidic | 3.7 ± 0.3 |  | 2.5 ± 0.2 |  | 2.3 ± 0.2 |  |

Means and SE values of the experiments presented in figures 8 and S8. Nc: number of cells, ne: number of experiments and nt: number of transformed pools from which the data have been derived.

# Supplemental references

1. Sievers F, Higgins DG. Clustal omega, accurate alignment of very large numbers of sequences. Methods in molecular biology 2014; 1079:105-16.

2. Ronquist F, Huelsenbeck JP. MrBayes 3: Bayesian phylogenetic inference under mixed models. Bioinformatics 2003; 19:1572-4.

3. Schultz J, Milpetz F, Bork P, Ponting CP. SMART, a simple modular architecture research tool: identification of signaling domains. Proc Natl Acad Sci USA 1998; 95:5857-64.

4. Parikh A, Miranda ER, Katoh-Kurasawa M, Fuller D, Rot G, Zagar L, et al. Conserved developmental transcriptomes in evolutionarily divergent species. Genome Biol 2010; 11:R35.

5. Gloeckner G, Lawal HM, Felder M, Singh R, Singer G, Weijer CJ, et al. The multicellularity genes of dictyostelid social amoebas. Nature communications 2016; 7:12085.

6. Baskaran S, Ragusa MJ, Boura E, Hurley JH. Two-site recognition of phosphatidylinositol 3-phosphate by PROPPINs in autophagy. Molecular cell 2012; 47:339-48.

7. Krick R, Busse RA, Scacioc A, Stephan M, Janshoff A, Thumm M, et al. Structural and functional characterization of the two phosphoinositide binding sites of PROPPINs, a beta-propeller protein family. Proceedings of the National Academy of Sciences of the United States of America 2012; 109:E2042-9.
